# ZBTB17/MIZ1 promotes peroxisome biogenesis by transcriptional regulation of PEX13

**DOI:** 10.1083/jcb.202407198

**Published:** 2025-04-17

**Authors:** Hongqin Liu, Xi Chen, Hanlin Wang, Guanglei Zhuang, Zheng-Jiang Zhu, Min Zhuang

**Affiliations:** 1 School of Life Science and Technology, ShanghaiTech University, Shanghai, China; 2 University of Chinese Academy of Sciences, Beijing, China; 3 Interdisciplinary Research Center on Biology and Chemistry, Shanghai Institute of Organic Chemistry, Chinese Academy of Sciences, Shanghai, China; 4 State Key Laboratory of Chemical Biology, Shanghai Institute of Materia Medica, Chinese Academy of Sciences, Shanghai, China; 5 Shandong Laboratory of Yantai Drug Discovery, Bohai Rim Advanced Research Institute for Drug Discovery, Yantai, China; 6 State Key Laboratory of Systems Medicine for Cancer, Department of Obstetrics and Gynecology, Shanghai Cancer Institute, Ren Ji Hospital, Shanghai Jiao Tong University School of Medicine, Shanghai, China; 7 Shanghai Key Laboratory of Gynecologic Oncology, Ren Ji Hospital, Shanghai Jiao Tong University School of Medicine, Shanghai, China; 8 Shanghai Key Laboratory of Aging Studies, Shanghai, China

## Abstract

Peroxisomes are integral metabolic organelles involved in both catabolic and anabolic processes in humans, with defects linked to diseases. The functions of peroxisomes are regulated at transcriptional, translational, and posttranslational levels. In this study, we employed the CRISPR/Cas9-based screening of a ubiquitin ligase library to identify regulators of human peroxisomes. We discovered that ZBTB17 (MIZ1) plays a role in regulating the import of proteins into peroxisomes. Independent of its ubiquitin ligase activity, ZBTB17/MIZ1 operates as a transcription factor to modulate the expression of key importer PEX13, influencing the localization of peroxisomal enzymes. Furthermore, metabolomic profiling reveals that knockdown of *ZBTB17* or *PEX13* results in similar metabolic alterations, with downregulated purine synthesis. Collectively, we identify ZBTB17 as a key regulator of peroxisomal protein import, thereby affecting peroxisomal function and nucleotide metabolism. Our findings provide insights into the multifaceted regulation of peroxisomes in complex human cells and shed light on the molecular mechanisms underlying ZBTB17’s role as a transcriptional regulator.

## Teaser

Peroxisomes are essential cellular organelles whose dysfunction leads to human diseases. Researchers identify ZBTB17 as a crucial regulator of peroxisome function through its control of protein import machinery. This discovery reveals a previously unknown regulatory mechanism of peroxisome biology and cellular metabolism.

## Introduction

Peroxisomes are dynamic, membrane-bound organelles that play crucial roles in numerous cellular metabolic reactions, including fatty acid β-oxidation, ether lipid and bile acid synthesis, cholesterol transport, reactive oxygen species (ROS) metabolism, and antiviral signaling (Chu et al., 2015; Dixit et al., 2010; Islinger et al., 2018; Smith and Aitchison, 2013). The size and quantity of peroxisomes can greatly vary among different species. Mammalian cells maintain peroxisome homeostasis through a balance between regulated biogenesis and degradation (Mahalingam et al., 2021). Many *PEX* genes have been characterized, encoding peroxin proteins that operate in various stages of peroxisome biogenesis. These include *PEX3*, *PEX19*, and *PEX16* for membrane formation (Höhfeld et al., 1991; Kim and Mullen, 2013; Matsuzaki and Fujiki, 2008; Shibata et al., 2004); *PEX1, 2, 5, 6, 7, 10, 12, 13, 14*, and *26* for matrix protein import (Dammai and Subramani, 2001; El Magraoui et al., 2012; Feng et al., 2022a; Meinecke et al., 2010; Platta et al., 2007; Titorenko and Rachubinski, 2000; Walton et al., 1995); and *PEX11* for peroxisome proliferation (Chang et al., 2015; Thomas et al., 2015). Mutations in *PEX* genes can result in peroxisome biogenesis disorders, whereby patients have reduced or no peroxisomes and often face early mortality (Francis et al., 1995; Gould and Valle, 2000; Zalckvar and Schuldiner, 2022). Dysfunctions in peroxisomes have also been associated with neurodegenerative disease, aging, cancer, and type 2 diabetes (Waterham et al., 2016; Zalckvar and Schuldiner, 2022).

Peroxisome homeostasis is highly regulated at different levels. Notably, the biogenesis of peroxisomes is known to be controlled at the transcriptional level. In the methylotrophic yeast *Pichia pastoris*, the transcription factor Mxr1p induces the expression of several *PEX* genes and metabolic enzymes (Lin-Cereghino et al., 2006). In rodent cells, the activation of the peroxisome proliferator-activated receptor (PPAR) transcription factor family induces the biogenesis and proliferation of peroxisomes (Vamecq and Latruffe, 1999). The biogenesis and degradation of peroxisomes are also tightly regulated by posttranslational modifications, with ubiquitination playing a significant role (Platta et al., 2007; Sargent et al., 2016; Zheng et al., 2022). Specifically, the PEX2/10/12 ubiquitin ligase complex is necessary for matrix protein import during peroxisome biogenesis (El Magraoui et al., 2012; Feng et al., 2022a). In contrast, overexpression of PEX2 triggers pexophagy (Sargent et al., 2016), a process leading to the programmed degradation of peroxisomes via autophagy. In addition, amino acid depletion or mTOR inhibition also induces pexophagy, but in a manner dependent on the ubiquitin ligase MARCH5 (Zheng et al., 2022). Therefore, ubiquitin ligases are instrumental in regulating both peroxisome biogenesis and degradation.

Genetic screening in yeast has identified numerous *PEX* genes important for peroxisome biogenesis (Distel et al., 1996), and these genes exhibit a high degree of conservation in mammalian cells. However, given the larger number of peroxisomes in human cells compared with the limited number typically found in yeast, it is expected that the regulation of peroxisomes in human cells is more complex. This complexity could extend to both the organelle level, through the regulation of peroxisome numbers, and the enzyme level by controlling the import of peroxisomal metabolic enzymes. Such regulatory mechanisms would allow the fine-tuning of peroxisome functions. In this study, we hypothesized that there might be other peroxisome regulators in human cells. To investigate this, we employed a sgRNA library targeting ubiquitin ligases to screen for potential peroxisome regulators using CRISPR/Cas9 technology. We identified and confirmed the regulatory role of ZBTB17 in peroxisome protein import. ZBTB17 possesses a domain architecture comprising both the BTB domain, which is essential for ubiquitin ligase activity, and zinc finger motifs, which are necessary for DNA binding in transcriptional regulation. Further characterization revealed ZBTB17 as a transcription factor that directly controls *PEX13* expression to regulate peroxisomal protein import, thereby influencing cellular metabolism.

## Results

### Identification of potential peroxisome regulators by the CRISPR/Cas9 screen

**Figure S1.**
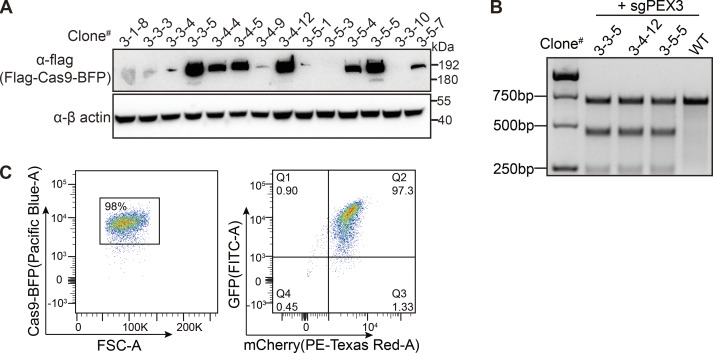
**Isolation of a single cell clone containing both Cas9 and the reporter. (A)** Cas9 expression level detected with anti-flag antibody in different clones. **(B)** Lentivirus-delivered sgRNA targeting the PEX3 gene in the indicated cell clones were assayed by T7E1 digestion showing Cas9 is active in the stable cell line. **(C)** Generation of Cas9 and EGFP/mCherry containing cell clone. Flow cytometry analysis of cell population expressing fluorescent proteins. Left panel: Forward scatter (FSC-A) versus Cas9-BFP fluorescence showing gating of BFP-positive healthy cells (98% of the total population). Right panel: Dual-parameter plot showing GFP (FITC-A) versus mCherry (PE-Texas Red-A) expression in the gated population. Source data are available for this figure: SourceData FS1.

**Figure 1.**
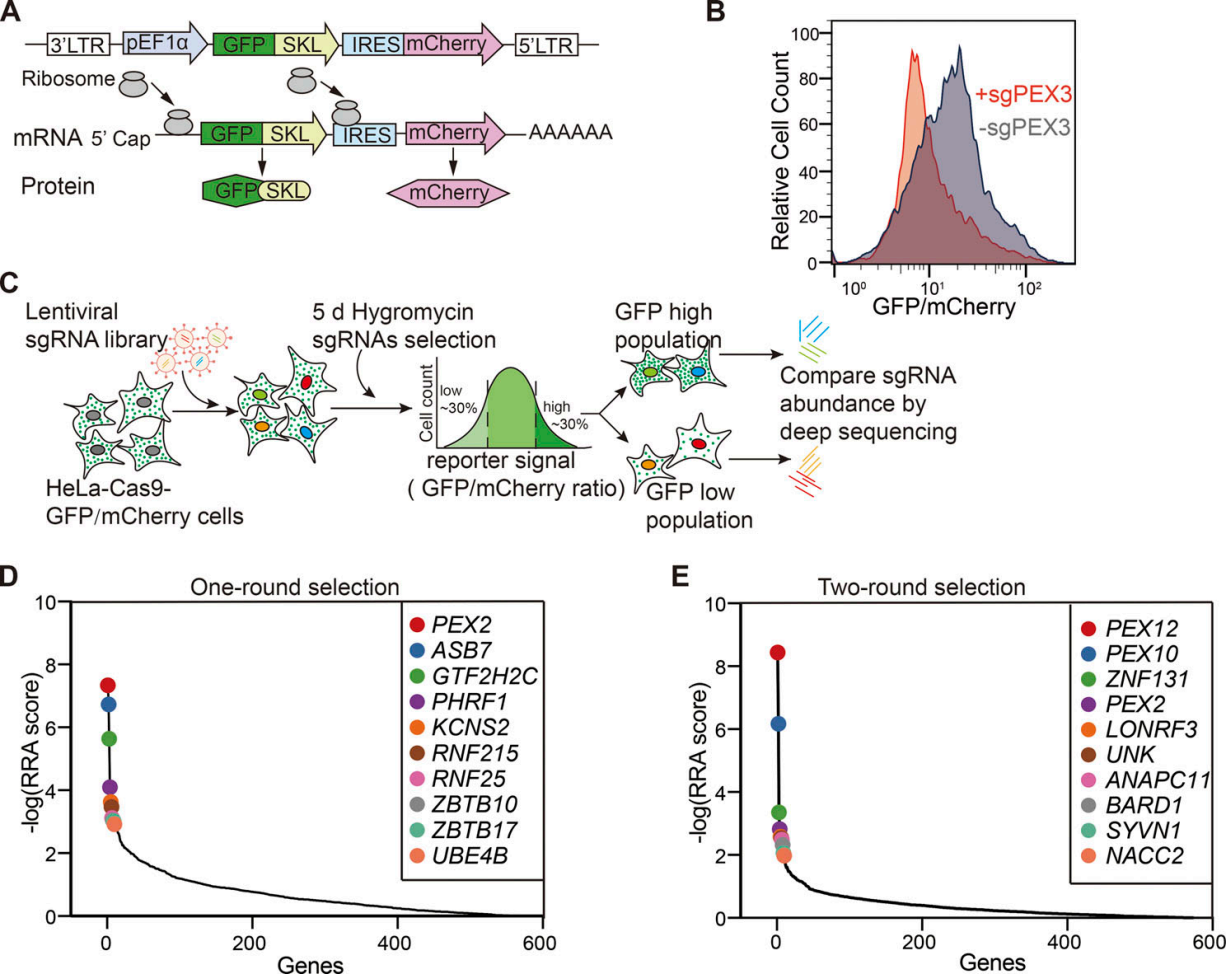
**Identification of peroxisome regulators using a dual-fluorescence reporter system coupled with a CRISPR screen. (A)** Diagram of the peroxisome reporter pEF1α–GFP(SKL)–IRES–mCherry. SKL stands for Ser–Lys–Leu fused to the C terminus of GFP; IRES stands for internal ribosome entry site. **(B)** Validation of the reporter cell line. HeLa–Cas9–poGFP/mCherry reporter cells were infected with the sgRNA targeting PEX3 for 48 h, and subsequently treated with puromycin for 2 days. GFP/mCherry signals were analyzed by flow cytometry. The reporter cells infected with a non-targeting sgRNA (sgNTC) were used as a control. **(C)** Schematics of the screening procedure. HeLa–Cas9–poGFP/mCherry cells were transduced with the ubiquitin ligase lenti-CRISPR library. 24 h after transduction, hygromycin was added to cells and maintained for 5 days. The surviving cells were sorted for the lowest and highest 30% of the GFP/mCherry ratio. sgRNAs were amplified from the extracted genomic DNA of each sample for deep sequencing. **(D)** Illustration of the top 10 candidate genes with the highest Robust Rank Aggregation (RRA) scores, calculated from the enrichment of sgRNAs in the GFP low (GFP/mCherry ratio at the bottom 30%) cells compared with GFP high (GFP/mCherry ratio at the top 30%) cells. The x-axis represents the ranked genes and the y-axis shows the negative log-transformed RRA scores. The black line shows the overall distribution of RRA scores for all genes. The colored dots highlight specific genes of interest (top hits) (Table S3). **(E)** Illustration of the top 10 candidate genes in the two-round sorting screen. RRA scores are calculated from the enrichment of sgRNAs in the sorted cells compared with unsorted cells (Table S3).

To perform the genetic screen in HeLa cells, we first generated a HeLa cell line stably expressing spCas9 (HeLa-Cas9). The expression of spCas9 in various clones was confirmed using anti-Flag immunoblots, and its activity was validated using the T7 assay (Fig. S1, A and B). To generate a peroxisome reporter, we design a construct containing both GFP–SKL (poGFP) and mCherry with an internal ribosome entry site (IRES) sequence in between (Fig. 1 A). SKL is a peroxisome-targeting peptide composed of three amino acids, Ser–Lys–Leu. GFP–SKL mainly localizes in peroxisomes while mCherry remains in the cytoplasm. The GFP/mCherry ratio can be used to monitor the change of peroxisomes in cells. We generated a stable cell line expressing GFP(SKL)–IRES–mCherry on the background of HeLa-Cas9 cells. The expression of both GFP–SKL and mCherry was confirmed with flow cytometry (Fig. S1 C), and we named this cell line HeLa–Cas9–poGFP/mCherry.

To validate HeLa–Cas9–poGFP/mCherry as a reliable peroxisome function reporter cell, we designed an sgRNA to target *PEX3* (Table S1). PEX3 is a peroxisomal protein required for the assembly of peroxisomal membrane proteins (PMPs) for the biogenesis of peroxisomes. *PEX3*-deficient cells lack functional peroxisomes. Flow cytometry analysis of cells in the presence of sgRNA targeting PEX3 (sgPEX3) revealed a significant decrease in GFP/mCherry signal compared with control cells (Fig. 1 B). This suggests that HeLa–Cas9–poGFP/mCherry can be utilized as a reporter for peroxisome function.

We screened a specialized CRISPR sgRNA library, in which there were 5,204 sgRNAs targeting 573 human genes (∼10 sgRNAs/each target) (Table S2). Each gene encodes a potential ubiquitin ligase, characterized by a RING domain, a HECT domain, or a cullin-interacting domain. Among those ubiquitin ligases, PEX2, PEX10, and PEX12 are three ubiquitin ligases that form a trimeric complex essential for peroxisome protein import (Feng et al., 2022b).

**Figure S2.**
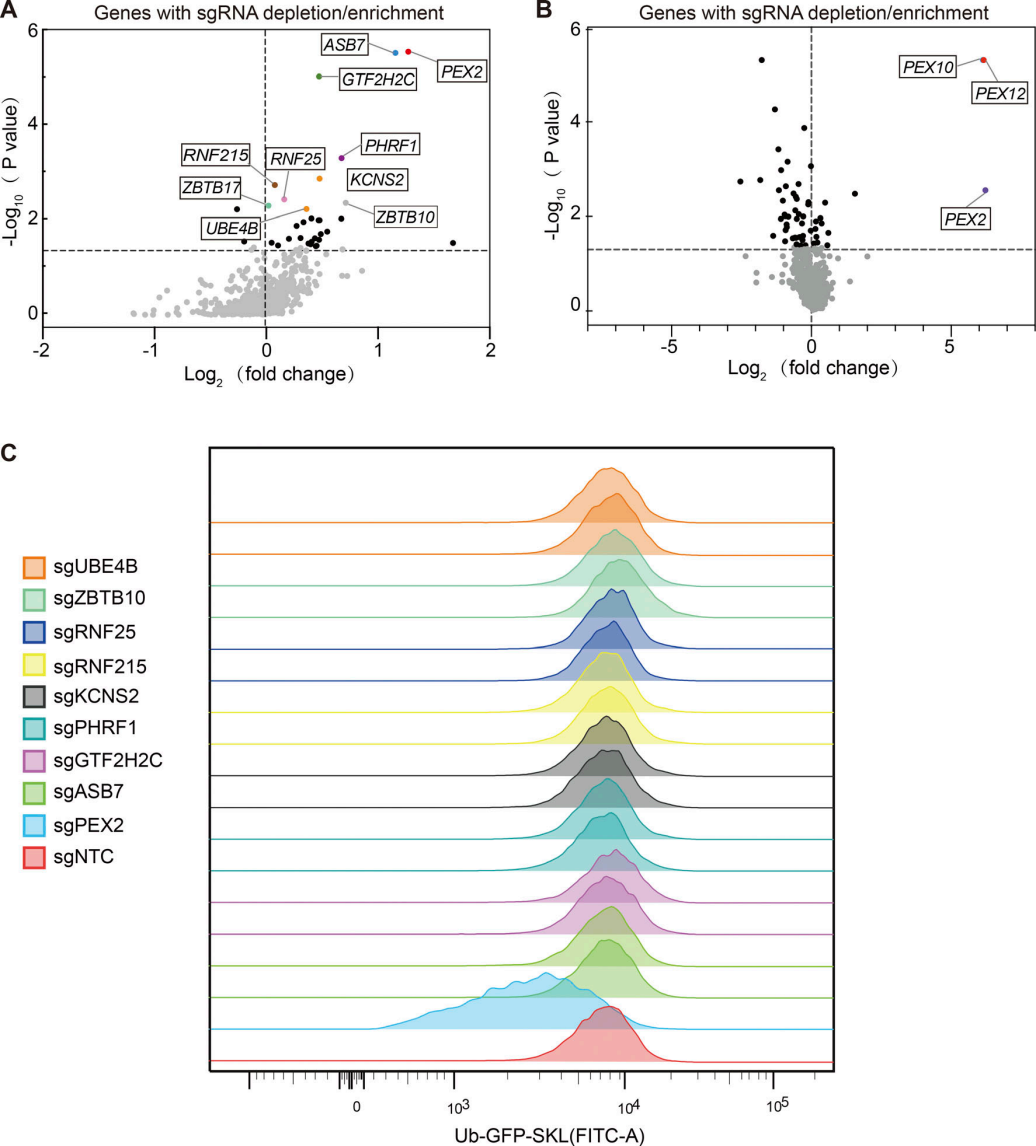
**Volcano plot illustrating the differential expression of sgRNAs (shown as the name of the targeting genes). (A)** Sequencing results from the one-round screen. The fold change indicates the enrichment of these sgRNAs in the lowest 30% of the GFP/mCherry cells compared with the highest 30% of the GFP/mCherry cells. The top candidate genes are highlighted. **(B)** Sequencing results from the two-round screen. The fold changes are calculated from the sgRNAs in the sorted cells compared with unsorted cells. **(C)** Flow cytometry analysis of cells expressing Ub–GFP–SKL using two different sgRNAs for each candidate gene knockout.

We applied two types of screening strategies with different stringencies, aiming to identify positive regulators. First, HeLa–Cas9–poGFP/mCherry cells were transfected with the sgRNA library, cultured with hygromycin selection for 5 days, and then harvested and subjected to flow cytometry sorting. Cells with GFP/mCherry ratio at the bottom 30% and top 30% were sorted and sequenced individually (Fig. 1 C). sgRNAs targeting positive regulators are expected to be enriched in the GFP low sample. Therefore, fold changes of sgRNAs (GFP low/GFP high) were analyzed and presented via Robust Rank Aggregation (RRA) score (Fig. 1 D and Table S3). Genes as the target of differentially expressed sgRNAs were presented in a volcano plot (Fig. S2 A and Table S4). Ubiquitin ligase PEX2, a well-known peroxisome transport regulator, ranks the highest in the screen (Fig. 1 D). However, PEX10 and PEX12, which are two ubiquitin ligases that form a trimeric complex with PEX2, did not appear as top-ranked hits in this one-round screen.

We also applied a more stringent two-round screening strategy. In this screen, cells with GFP fluorescence at the bottom 30% were sorted and cultured for another 6 days, followed by another round of sorting cells with the bottom 30% GFP intensity. The cells were then harvested for sgRNA sequencing. sgRNAs that target *PEX2*, *PEX10*, and *PEX12* were all highly enriched in the sorted sample compared with unsorted cells (Fig. 1 E). However, since PEX2, PEX10, and PEX12 are all essential for peroxisomal protein import, they dominated in the stringent screen (Fig. S2 B). We rationalize that potential peroxisome regulators are more likely to be present in the less stringent one-round screening.

### ZBTB17 regulates peroxisome protein import

To validate the screening hits, we generated another reporter cell stably expressing Ub–GFP–SKL, in which ubiquitin is fused to the N terminus of GFP–SKL with Gly76 mutated to valine to prevent deubiquitination (Zheng et al., 2022, 2025). Ub–GFP–SKL is degraded fast by the ubiquitin-proteasome degradation system in the cytosol (Dantuma et al., 2000), while it is protected in peroxisomes once imported. With this design, minimal cytosolic Ub–GFP–SKL can be detected, further eliminating non-peroxisomal GFP background.

**Figure 2.**
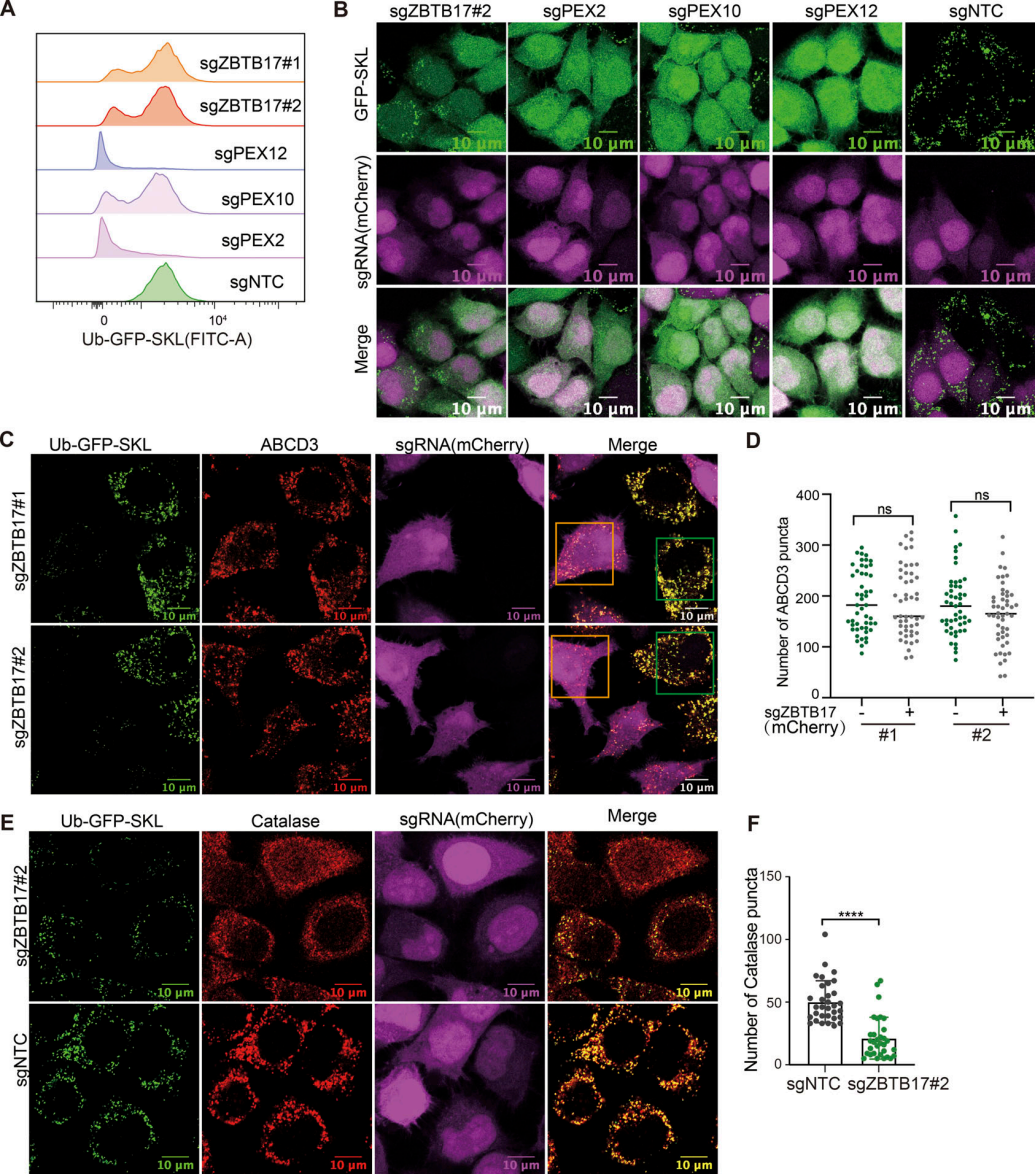
**ZBTB17 regulates the translocation of peroxisomal matrix proteins. (A)** ZBTB17 knockout reduces Ub–GFP–SKL levels. Flow cytometry analysis of GFP signals in HeLa cells stably expressing Ub–GFP–SKL (UGS cells in brief). sgZBTB17, sgPEX2, sgPEX10, sgPEX12, or sgNTC were used to knock out indicated genes. sgZBTB17#1 and sgZBTB17#2 are two different sgRNA sequences. Cells were analyzed 4 days after transfection. **(B)** ZBTB17 knockout causes the cytosolic retention of GFP–SKL. Fluorescence microscopy images showing the cellular location of GFP–SKL and mCherry in HeLa cells stably expressing GFP–SKL and transfected with different sgRNAs (sgZBTB17, sgPEX2, sgPEX10, sgPEX12). sgRNAs were co-expressed with mCherry. **(C)** Representative immunofluorescence microscopy images of Ub–GFP–SKL HeLa cells transfected with indicated sgRNAs and stained for ABCD3. Orange box, sgRNA transfected cell, mCherry positive; Green box, untransfected cell, mCherry negative. **(D)** Quantification of numbers of ABCD3 puncta in >90 cells for C. Values are mean ± SD, n.s., not significant, by two-tailed Student’s *t* test. **(E)** ZBTB17 knockout causes the cytosolic retention of catalase. Immunofluorescence microscopy images of Ub–GFP–SKL HeLa cells transfected with indicated sgRNAs and stained for catalase. Scale bars, 10 µm. **(F)** Quantification of Catalase puncta in E. Values are mean ± SD, calculated using 32 cells. ****P < 0.0001 by two-tailed Student’s *t* test.

Targeting the top 10 hits from the screening, we transfected cells with individual sgRNAs targeting each gene and examined the fluorescence intensity of Ub–GFP–SKL. Among all validated genes, knocking out *ZBTB17* (also known as *MIZ-1*) significantly reduces the Ub–GFP–SKL intensity in cells, similar to what was observed for *PEX2*, *PEX10*, and *PEX12* (Fig. 2 A). All other hits do not affect the Ub–GFP signal when tested individually (Fig. S2 C). The effect of *ZBTB17* knockout was further confirmed in cells expressing GFP–SKL (Fig. 2 B). The presence of sgRNA is indicated by the coexpressed mCherry. In the presence of a non-targeting sgRNA control (sgNTC), GFP–SKL mainly localizes within peroxisomes, showing GFP dots. In the presence of sgRNA targeting *PEX2*, *PEX10*, or *PEX12*, the peroxisome protein import is impaired, resulting in the distribution of GFP–SKL in the cytosol. With the sgRNA targeting *ZBTB17*, the cells also display cytosolic GFP–SKL distribution, suggesting peroxisome defects (Fig. 2 B).

The abnormal GFP–SKL distribution can be explained by either defective GFP–SKL import or the loss of peroxisomes. Therefore, we examined peroxisome numbers. We transfected Ub–GFP–SKL–containing cells with sgRNA targeting *ZBTB17* (mCherry positive) with low transfection efficiency so that the untransfected cells (mCherry negative) can be used as the control group. The peroxisome membrane protein marker ABCD3 (also referred to as PMP70) was stained. The number of peroxisomes was determined by counting ABCD3-positive specks, and statistical analysis revealed no change in peroxisome numbers in *ZBTB17* knockout cells (Fig. 2, C and D).

**Figure S3.**
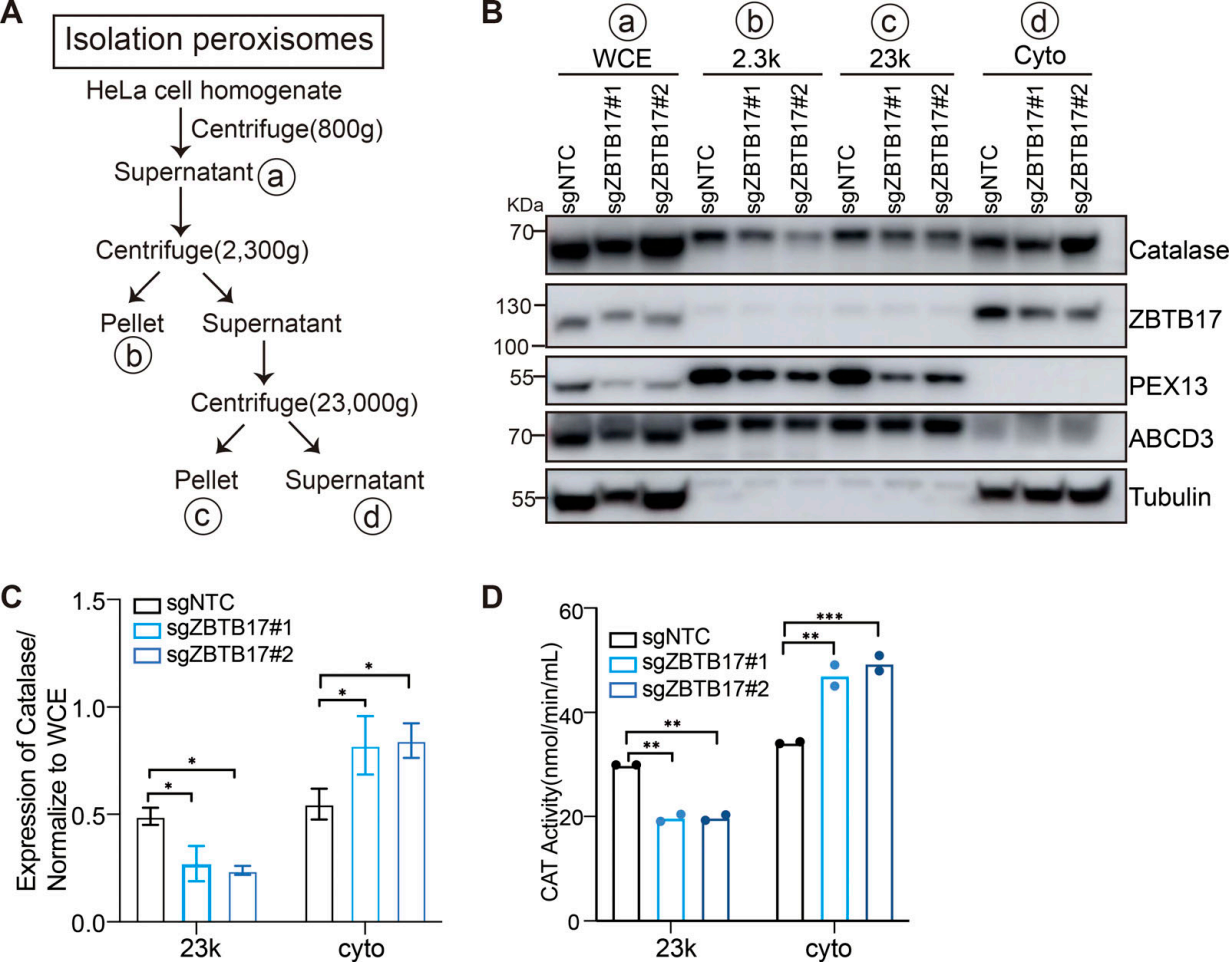
**Catalase is redistributed in ZBTB17 deficient cells. (A)** Flowchart of the peroxisome purification procedure. **(B)** Western blot analysis of catalase in different fractions in cells with or without ZBTB17 knockout. Cellular fractionation experiments were performed using stepwise centrifugation, and equal volume of samples with each fraction isolated from cells was analyzed with IB using the indicated antibodies. WCE: whole-cell extraction; 2.3K: pellet after centrifugation at 2,300 × *g*; 23K: the major peroxisome fraction, pellet after centrifugation at 23,000 × *g*; Cyto: supernatants after centrifugation at 23,000 × *g*. **(C)** Catalase levels in various fractions of distinct samples were quantified and normalized to WCE using ImageJ densitometric quantification. Values are mean ± SD, *n* = 3 independent experiments. *, P < 0.05; by two-way ANOVA multiple comparisons test. **(D)** Catalase activity in various fractions of distinct samples was measured. **, P < 0.01; ***, P < 0.001; by two-way ANOVA multiple comparisons test (*n* = 2). Source data are available for this figure: SourceData FS3.

In addition to GFP–SKL, the cellular localization of catalase, an endogenous peroxisome matrix enzyme, was investigated. Catalase is widely distributed in the cytoplasm in the presence of sgRNA targeting *ZBTB17* (Fig. 2 E), and less catalase puncta can be observed (Fig. 2 F). We also conducted a cellular fractionation experiment (Fig. S3 A). In *ZBTB17* knockout cells, less catalase was detected in the peroxisome-enriched fraction (23k), accompanied by a decrease of PEX13 level in these fractions (Fig. S3 B). Catalase activity was measured, suggesting a reduced enzyme content in peroxisomes and an increase in the cytosol in *ZBTB17* knockout cells (Fig. S3 D). ABCD3 levels remained consistent in different cells (Fig. S3 B), aligning with the observation of unchanged peroxisome numbers. Therefore, ZBTB17 regulates peroxisome protein import.

### The ubiquitin ligase activity of ZBTB17 is not essential for peroxisome protein import

**Figure 3.**
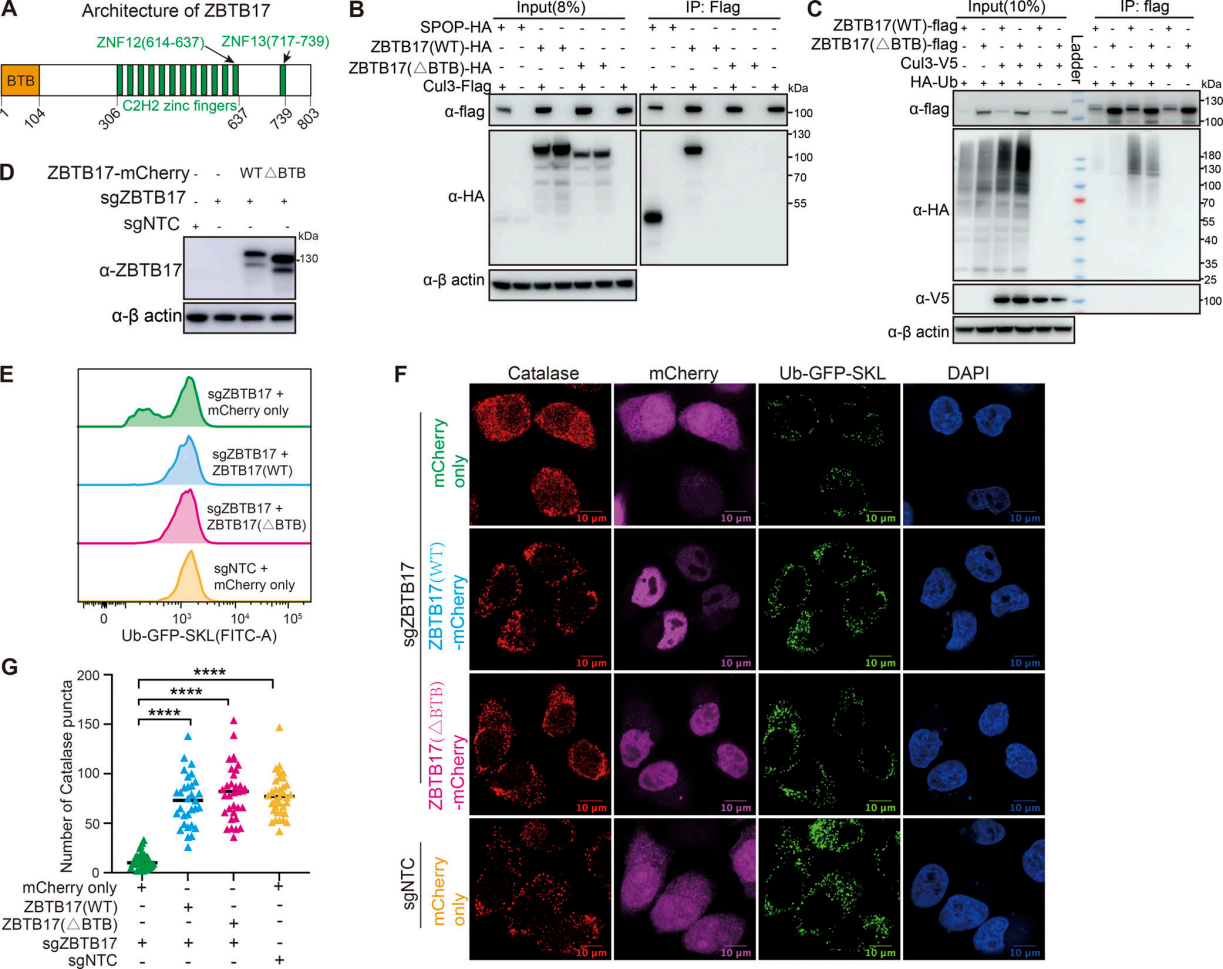
**The BTB domain of ZBTB17 is not required for peroxisome protein import. (A)** Schematics of the domain arrangement of ZBTB17. **(B)** Coimmunoprecipitation of ZBTB17(WT)-HA, ZBTB17(∆BTB)-HA, or SPOP-HA with Cul3-Flag in HEK293T cells. HEK293T cells were cotransfected ZBTB17-HA, ZBTB17(∆BTB)-HA, or SPOP-HA along with Cul3-Flag for 24 h and subsequently treated with 10 µM MG132 for 4 h. Cell lysates were then subjected to immunoprecipitation using an anti-Flag antibody, followed by immunoblotting with the indicated antibodies. **(C)** Ubiquitination assay of ZBTB17 in cells co-transfected with HA-Ub, Cul3-V5, ZBTB17(WT)-Flag, or ZBTB17(∆BTB)-Flag. HEK293T cells were transfected with the mentioned plasmids for 48 h. Cell lysates were immunoprecipitated using an anti-Flag antibody and then analyzed by immunoblotting with the respective antibodies. **(D)** Immunoblotting analysis of ZBTB17-mCherry protein expression. **(E)** Analysis of Ub–GFP–SKL signal in ZBTB17 knockout UGS cells. ZBTB17 was eliminated from UGS cells using sgRNA. Following infection with ZBTB17 (WT)-mCherry, ZBTB17 (∆BTB)-mCherry, or mCherry-only virus for 48 h, the Ub–GFP–SKL signal was assessed via flow cytometry. **(F)** Immunofluorescence (IF) analysis of catalase and Ub–GFP–SKL. ZBTB17 was eliminated from UGS cells using sgRNA. Following this, cells were infected with ZBTB17 (WT)-mCherry, ZBTB17 (∆BTB)-mCherry, or mCherry-only virus for 48 h. The scale bar represents 10 µm. **(G)** Quantification of catalase puncta in UGS cells. Calculations were made using data from E (>30 cells for each condition). ****P < 0.0001, by two-tailed Student’s *t* test. Source data are available for this figure: SourceData F3.

ZBTB17 contains a BTB domain at the N terminus and 13 zinc fingers (ZNFs) at the C terminus (Fig. 3 A). It is included in the screening library as a ubiquitin ligase because the BTB domains, in general, bind Cul3 to form BTB/Cul3 ubiquitin ligases. ZBTB17 also functions as a transcription factor, but mainly via the C terminal ZNFs. We first sought to determine whether ZBTB17 plays a role as a ubiquitin ligase in peroxisome protein import.

To address if ZBTB17 forms an active ubiquitin ligase with Cul3, we examined the interaction between ZBTB17 and Cul3. Exogenously expressed ZBTB17, but not ZBTB17 (ΔBTB), associates with Flag-tagged Cul3 (Fig. 3 B). However, the interaction between ZBTB17 and Cul3 appears significantly weaker than that of the classic Cul3-binding BTB protein SPOP and Cul3, as shown by the reduced immunoprecipitation of ZBTB17-HA with Cul3-Flag. While endogenous Cul3 levels produced minimal ZBTB17 self-ubiquitination, Cul3 overexpression enhanced this process (Fig. 3 C). The BTB-deleted variant showed significantly reduced ubiquitination compared with wild-type ZBTB17, suggesting that the BTB domain is necessary for effective ubiquitin ligase function under normal cellular conditions.

To address if the BTB domain is required for normal Ub–GFP–SKL signals in cells, we reintroduced wildtype ZBTB17 or ZBTB17(ΔBTB) into *ZBTB17* knockout cells (Fig. 3 D). Full-length ZBTB17 reconstitutes the GFP signal as expected. The expression of ZBTB17(ΔBTB) is also sufficient to recover the GFP signal (Fig. 3 E), suggesting the BTB domain of ZBTB17 is not a major contributor to peroxisome regulation. The import of Ub–GFP–SKL and catalase into peroxisomes were recovered when wildtype ZBTB17 or ZBTB17(ΔBTB) was expressed in *ZBTB17* knockout cells (Fig. 3, F and G). These data suggest that although ZBTB17 may act as a ubiquitin ligase, its ligase activity is not essential for normal peroxisome import.

### ZBTB17 regulates *PEX13* transcription

In addition to its role as a ubiquitin ligase, ZBTB17 has been characterized for its role as a transcriptional regulator (Kosan et al., 2010; Peukert et al., 1997; Wiese et al., 2013). ZBTB17 predominantly localizes to the cell nucleus (Fig. 3 F), which aligns with its role as a transcription factor. Therefore, we investigated ZBTB17 as a transcriptional regulator for peroxisome protein import.

**Figure S4.**
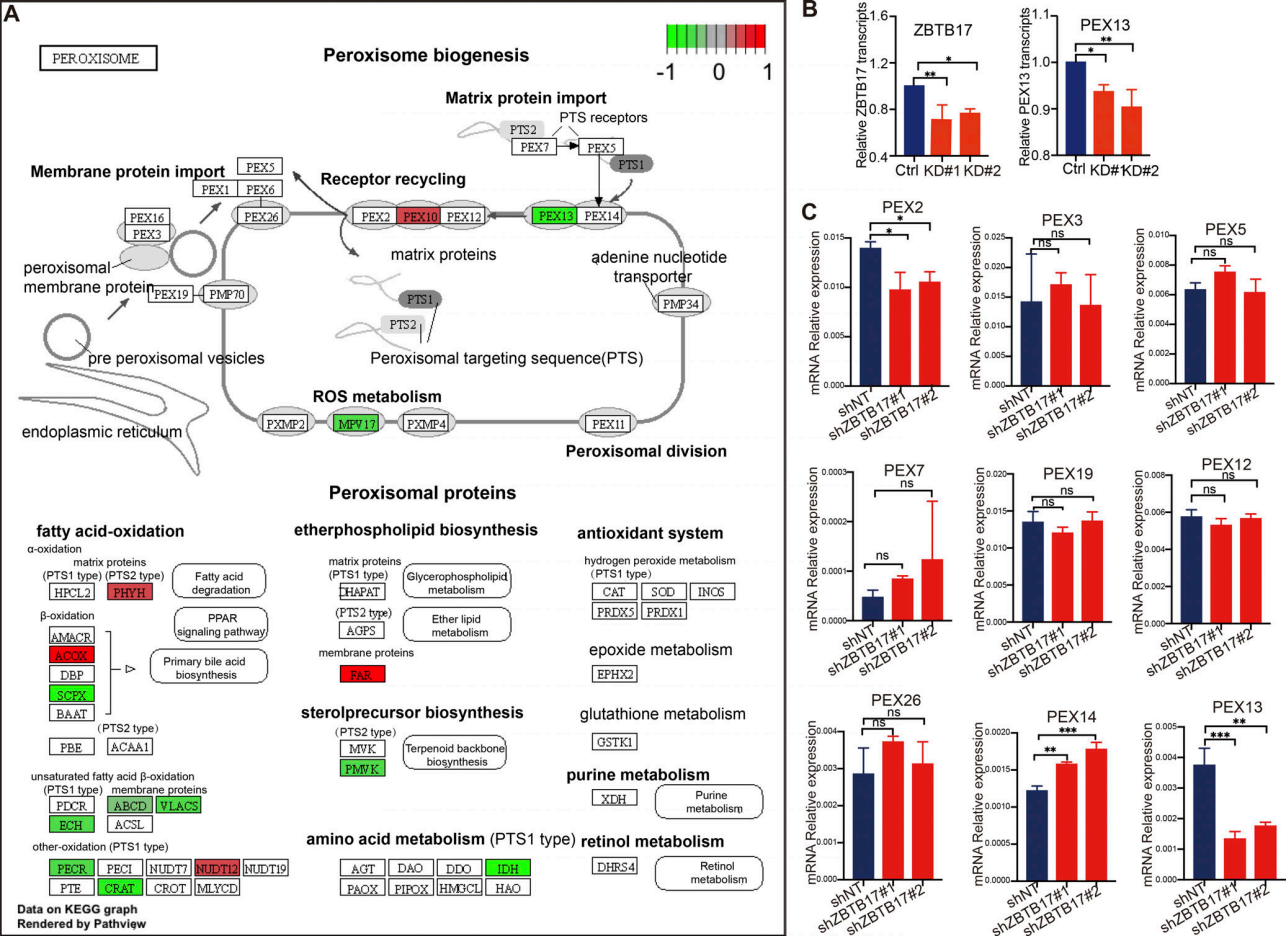
**Other peroxisome-related genes with altered expression. (A)** Peroxisome-related genes with altered expression are highlighted in KEGG (Kyoto Encyclopedia of Genes and Genomes) pathway. Red, upregulated genes; green, downregulated genes. This visualization was conducted using the R package “pathview” (Luo and Brouwer, 2013) on KEGG graphs (P.adjust < 0.05). **(B)** Relative levels of ZBTB17 and PEX13 transcripts in ZBTB17 knockdown and wild-type HeLa cells, as determined from RNA-seq data. Values are mean ± SD, *n* = 5 independent experiments. **(C)** Quantitative PCR (Q-PCR) analysis of the mRNA levels of peroxisome transport genes in ZBTB17 knockdown and wild-type HeLa cells. Values are mean ± SD, *n* = 3. Statistically significant differences are indicated (P < 0.05*; P < 0.01**; P < 0.0001***; n.s., not significant [P > 0.05]), by two-tailed Student’s *t* test.

**Figure 4.**
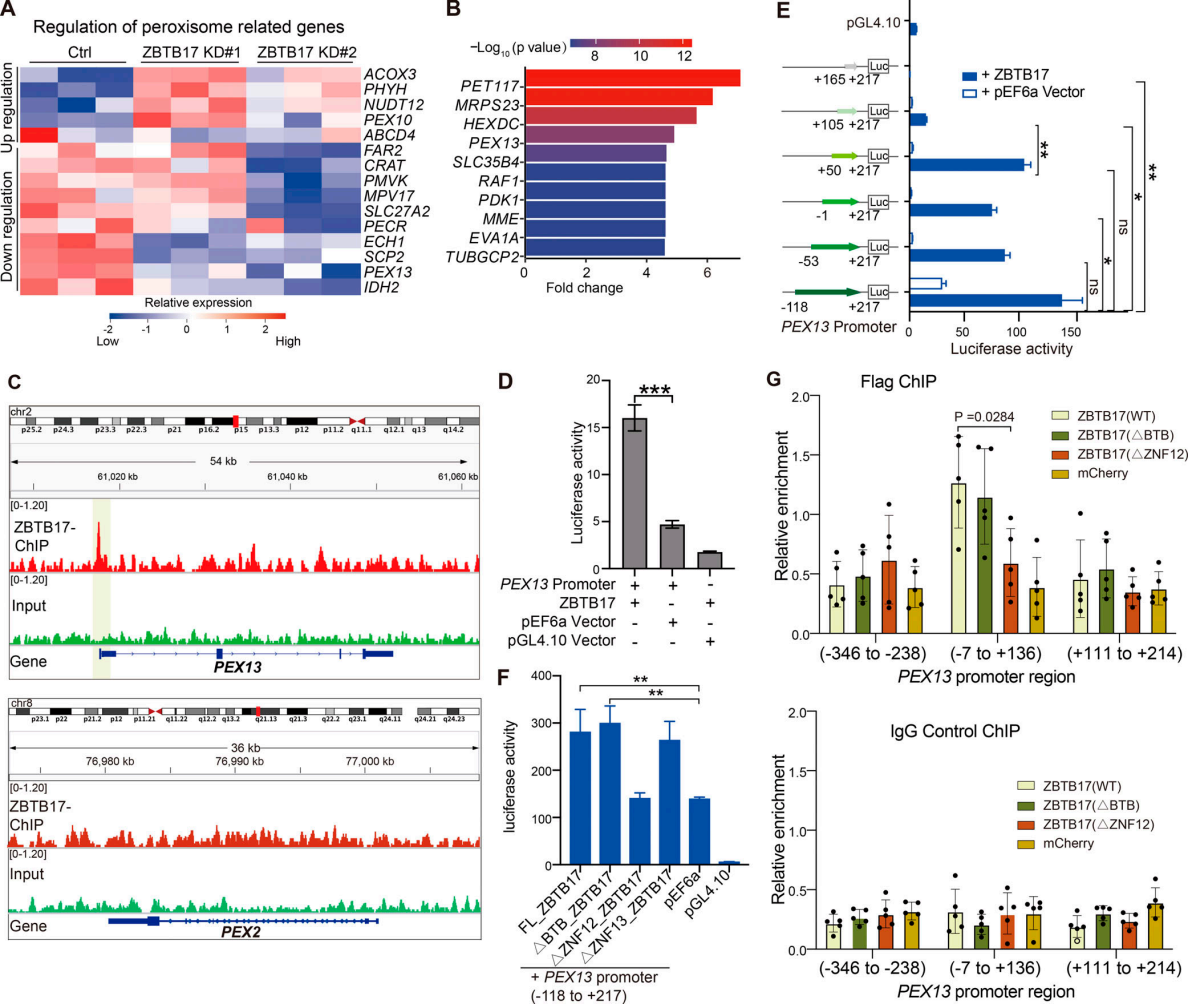
**ZBTB17 regulates PEX13 transcription directly by associating with the PEX13 promoter region. (A)** Heat map of differentially expressed peroxisomal genes in response to ZBTB17 knockdown. HeLa cells were infected with ZBTB17 shRNA (shZBTB17) or non-targeting shRNA (shNT) for 4 days and were subjected to RNA-seq analysis. Lower and higher levels of expression are presented in shades of blue and red, respectively (fold change >1.3; P.adjust < 0.05). **(B)** Top differentially bound genes identified by ChIP–seq analysis, ranked by fold change on the x-axis. The bars are colored according to statistical significance (−log_10_[P value] scale). The list is filtered for genes that are also downregulated in RNA-seq data (Fig. 1 D) to only include the differentially expressed genes. **(C)** ChIP-seq analysis revealed ZBTB17 binding at the PEX13 promoter in HeLa cells using PEX2 promoter as a reference. **(D)** Luciferase assay demonstrating the interaction of ZBTB17 with the PEX13 promoter. HEK293T cells were co-transfected with pGL4.10- PEX13 promoter (−1,886 to +115)- dual luciferase reporter and pEF6a-PEX13 for 24 h. Luminescence was quantified using a Luminescence Counter. Empty pGL4.10 and pEF6 vectors were used for controls. Values are mean ± SD, *n* = 3 independent experiments. ***, P < 0.001; by two-tailed Student’s *t* test. **(E)** Luciferase assay to map ZBTB17 binding regions on PEX13. The PEX13 promoter sequence (335 bp) was truncated into shorter fragments and cloned into the luciferase reporter vector. Luminescence was quantified in the presence or absence of con-transfected ZBTB17. Values are mean ± SD, *n* = 3 independent experiments. *, P < 0.05; **, P < 0.01; n.s., not significant, by two-tailed Student’s *t* test. **(F)** Luciferase assay to map PEX13 promoter binding regions on ZBTB17. ∆BTB, ∆ZNF12, ∆ZNF13 are ZBTB17 with the BTB (residues 1–104), ZNF12 (residues 614–637), or ZNF13 (residues 717–739) removed individually. Values are mean ± SD, *n* = 3 independent experiments. **, P < 0.01, by two-tailed Student’s *t* test. **(G)** ChIP-qPCR analysis validates the binding of ZBTB17 to the PEX13 promoter region. 3xFlag tagged ZBTB17(WT), ZBTB17(∆BTB), ZBTB17(∆ZNF12), or mCherry were expressed in HeLa cells for 48 h, and cells were subjected to ChIP assays using either anti-Flag antibodies or IgG control. Purified DNA was analyzed by quantitative real-time PCR amplified for different promoter regions of PEX13, O1 indicates the −346 to −238, O2 indicates the −7 to +136, and O3 indicates the +111 to +214. Values are mean ± SD, *n* = 5 independent experiments. Statistically significant differences are indicated (P = 0.0284) by paired *t* test.

HeLa cells were stably transfected with either a non-targeting shRNA or a shRNA specifically targeting *ZBTB17*. Transcriptome analysis based on RNA sequencing was carried out in HeLa cells with or without *ZBTB17* knockdown. A comparative analysis of gene expression yields a total of 3,178 differentially expressed genes (Table S5). Remarkably, among these genes, 15 genes associated with the peroxisome pathway were identified and their roles in peroxisome function are highlighted in the KEGG (Kyoto Encyclopedia of Genes and Genomes) pathway (Fig. S4). The heatmap displays a significant alteration in the expression of multiple peroxisome-related genes in cells with *ZBTB17* knockdown (Fig. 4 A). Specifically, five genes exhibited upregulated expression, while 10 genes displayed downregulated expression. Notably, *PEX13*, one of the key peroxins that controls the peroxisomal protein import, has been downregulated. The relative *ZBTB17* and *PEX13* transcripts in cells were derived from the RNA-seq data, confirming the downregulation of *PEX13* (Fig. S4 B).

PEX13 interacts with other peroxins, such as PEX5 and PEX14, forming a dynamic protein complex that aids in the translocation of proteins across the peroxisomal membrane (Barros-Barbosa et al., 2019; Gao et al., 2022; Meinecke et al., 2010; Ravindran et al., 2023). In addition to PEX13, peroxisomal matrix protein translocation also relies on PEX2, PEX5, PEX7, PEX10, PEX12, PEX14, and PEX26. Therefore, we examined the transcriptional level of these import-related genes individually by real-time RT-PCR (reverse transcription-polymerase chain reaction) in *ZBTB17* knockdown cells (Fig. S4 C). Most gene transcripts are not affected by *ZBTB17* knockdown, while the mRNA level of *PEX13* is significantly downregulated. *PEX2* expression is slightly reduced, but not to the extent of *PEX13*.

To further confirm the role of ZBTB17 as a transcriptional regulator, we mapped the associated DNA sequence by chromatin-immunoprecipitation (Ch-IP). By combining ChIP-seq and RNA-seq data, a few key genes are identified as potential ZBTB17 direct targets (Fig. 4 B). *PEX13* ranks high, and the ChIP-seq data also revealed that ZBTB17 binds specifically to the promotor region (nt −118 to +217) of *PEX13* (Fig. 4 C). However, other peroxisomal genes, such as *PEX2*, were not identified, suggesting that *PEX13* is the primary target gene and the others are likely being regulated indirectly.

To confirm *PEX13* as a direct target of ZBTB17, we performed a luciferase assay. In 293T cells, inserting the *PEX13* promoter into a luciferase vector induced luciferase expression, which was further enhanced by exogenous ZBTB17 (Fig. 4 D). To narrow down the ZBTB17 binding region on the *PEX13* promoter, we performed a systematic mapping. With the 335 bp sequence covered by ChIP-seq, the presence of ZBTB17 significantly induced luciferase expression. Systematic truncations identified that NT +50 to +105 on the *PEX13* promoter region are critical for ZBTB17 transactivity (Fig. 4 E). ZBTB17 contains 13 ZNFs, and it was previously reported that ZNF12 and ZNF13 are important for DNA binding (Boisvert et al., 2022; Peukert et al., 1997; Tremblay et al., 2016). We individually deleted ZNF12 and ZNF13 to investigate *PEX13* transcriptional regulation. The ΔZNF12 mutant is defective to induce luciferase activity, suggesting that ZBTB17 binds *PEX13* promoter via the ZNF12 motif (Fig. 4 F). Consistent with the luciferase assay, exogenously expressed ZBTB17(*ΔBTB*) binds to the endogenous PEX13 promoter while ZBTB17(ΔZNF12) failed to bind (Fig. 4 G).

Some BTB domains also bind DNA, as suggested in other BTB domain-containing proteins, such as BCL6 and PLZF (Stogios et al., 2005). However, the truncation of the ZBTB17 BTB domain had no effect on *PEX13* transcription (Fig. 4 F). This result is also consistent with the observation that ZBTB17(ΔBTB) can rescue peroxisome protein import in *ZBTB17* KO cells (Fig. 3 E).

### ZBTB17 regulates peroxisome protein import mainly via *PEX13*

**Figure 5.**
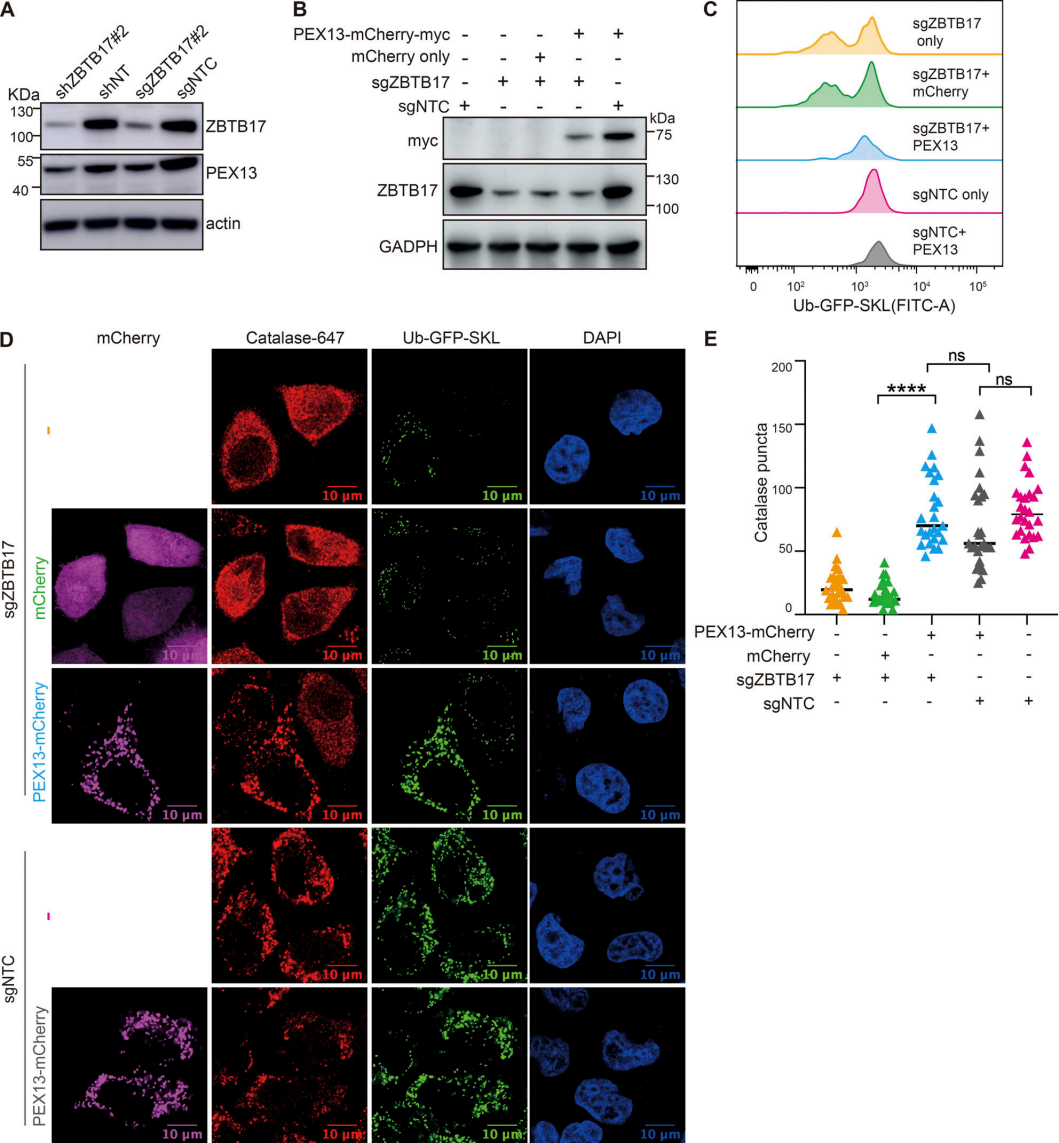
**ZBTB17 regulates peroxisome protein import via PEX13. (A)** Immunoblotting (IB) of PEX13 and ZBTB17 in HeLa cells followed by shRNA or sgRNA/Cas9 targeting ZBTB17. **(B)** Immunoblotting (IB) showing the corresponding protein expression levels in C. **(C)** Flow cytometry analysis of Ub–GFP–SKL in ZBTB17 knockout HeLa cells expressing either PEX13–mCherry–myc or mCherry for 48 h. **(D)** Representative immunofluorescence images showing the localization of catalase in corresponding conditions in B. Scale bars represent 10 µm. **(E)** Quantification of the number of catalase puncta in D. Calculated using >25 cells/sample. ****P < 0.0001; n.s., not significant, by two-tailed Student’s *t* test. Source data are available for this figure: SourceData F5.

**Figure S5.**
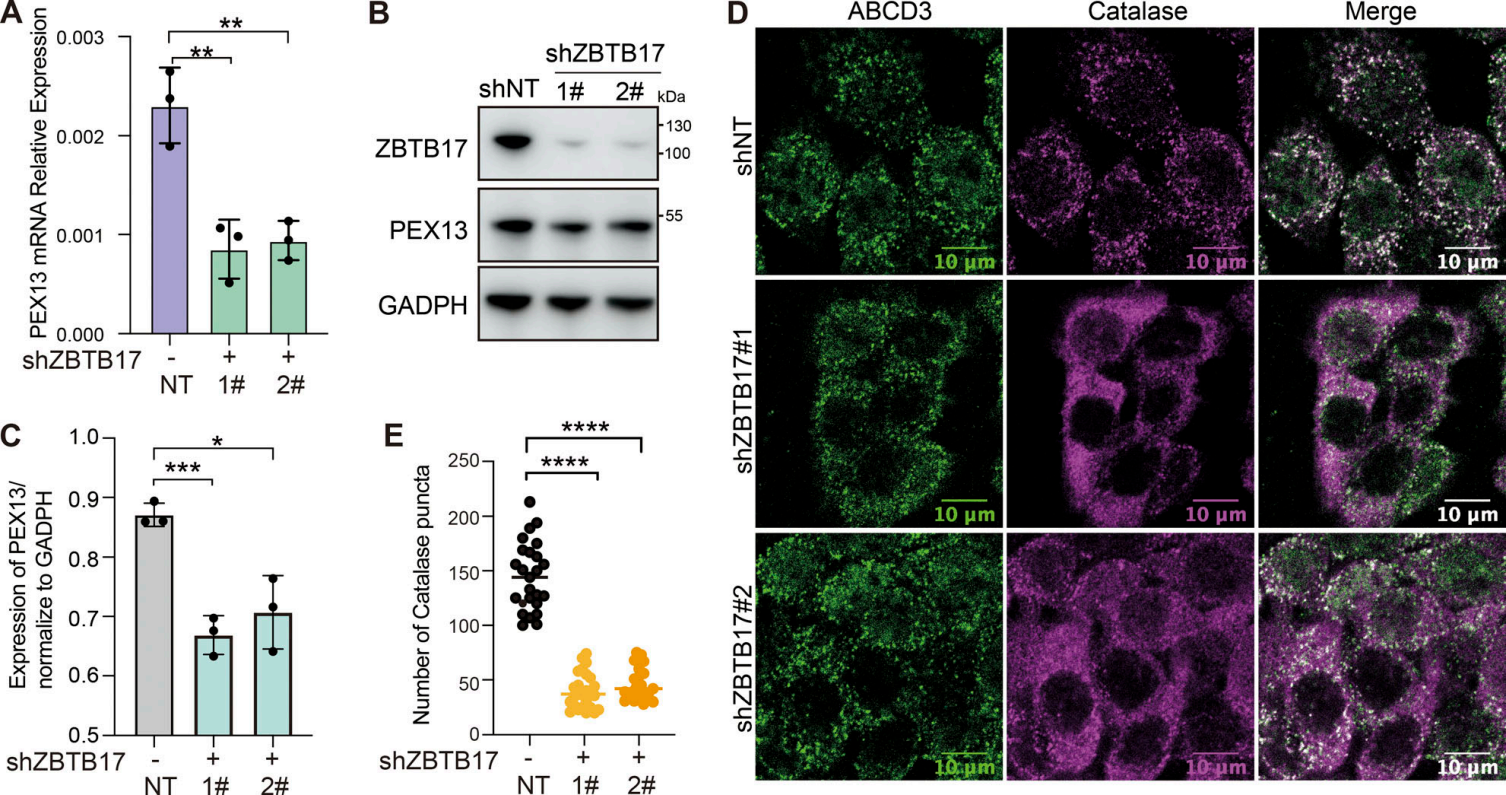
**ZBTB17 regulates the translocation of peroxisomal matrix proteins in HCT116 cells. (A)** RT-qPCR analysis of PEX13 mRNA expression in wild-type and ZBTB17-knockdown HCT116 cells. Values are mean ± SD, *n* = 3. **, P < 0.01. **(B)** Immunoblotting (IB) of PEX13 and ZBTB17 in HCT116 cells following ZBTB17 knockdown. **(C)** Densitometric quantification of PEX13 protein levels from B. Values are mean ± SD, *n* = 3 independent experiments. *, P < 0.05; ***, P < 0.001. **(D)** Representative immunofluorescence images showing catalase and ABCD3 localization in control and ZBTB17-knockdown HCT116 cells. **(E)** Quantification of catalase-positive puncta per cell from D. *n* = 25 cells. ****P < 0.0001. All statistical analyses were performed using two-tailed Student’s *t* test. Source data are available for this figure: SourceData FS5.

Consistent with the mRNA level, the protein level of PEX13 is also significantly decreased in HeLa cells with *ZBTB17* knockdown (Fig. 5 A). Similarly, in HCT116 cells, we also observed the downregulation of PEX13 mRNA and protein (Fig. S5, A–C) and the enhanced cytosolic distribution of catalase (Fig. S5, D and E) when ZBTB17 is knocked down.

As PEX13 is one of the key players for peroxisomal protein import, we sought to investigate whether the restoration of PEX13 protein level alone is adequate to re-establish peroxisomal protein import. In Ub–GFP–SKL cells, *ZBTB17* were knocked out with sgRNA, mCherry fused PEX13 or mCherry alone were introduced into these cells, and the expression of ZBTB17 and PEX13 in each sample was confirmed by western blots (Fig. 5 B). The Ub–GFP signal was quantified in each sample as an indicator of peroxisomal import. GFP signal was decreased in *ZBTB17* knockout cells but was restored upon exogenous expression of PEX13–mCherry, while mCherry alone had no effect on the GFP signal (Fig. 5 C). Similarly, we examined the localization of Ub–GFP–SKL and catalase under these conditions. The exogenous expression of PEX13 alone is adequate to restore peroxisomal import in the absence of ZBTB17 (Fig. 5, D and E). Despite the downregulated mRNA expression of *PEX2* (Fig. S4 C), these results again suggest that *PEX13* is the primary target of ZBTB17 in regulating peroxisomal import.

**Figure 6.**
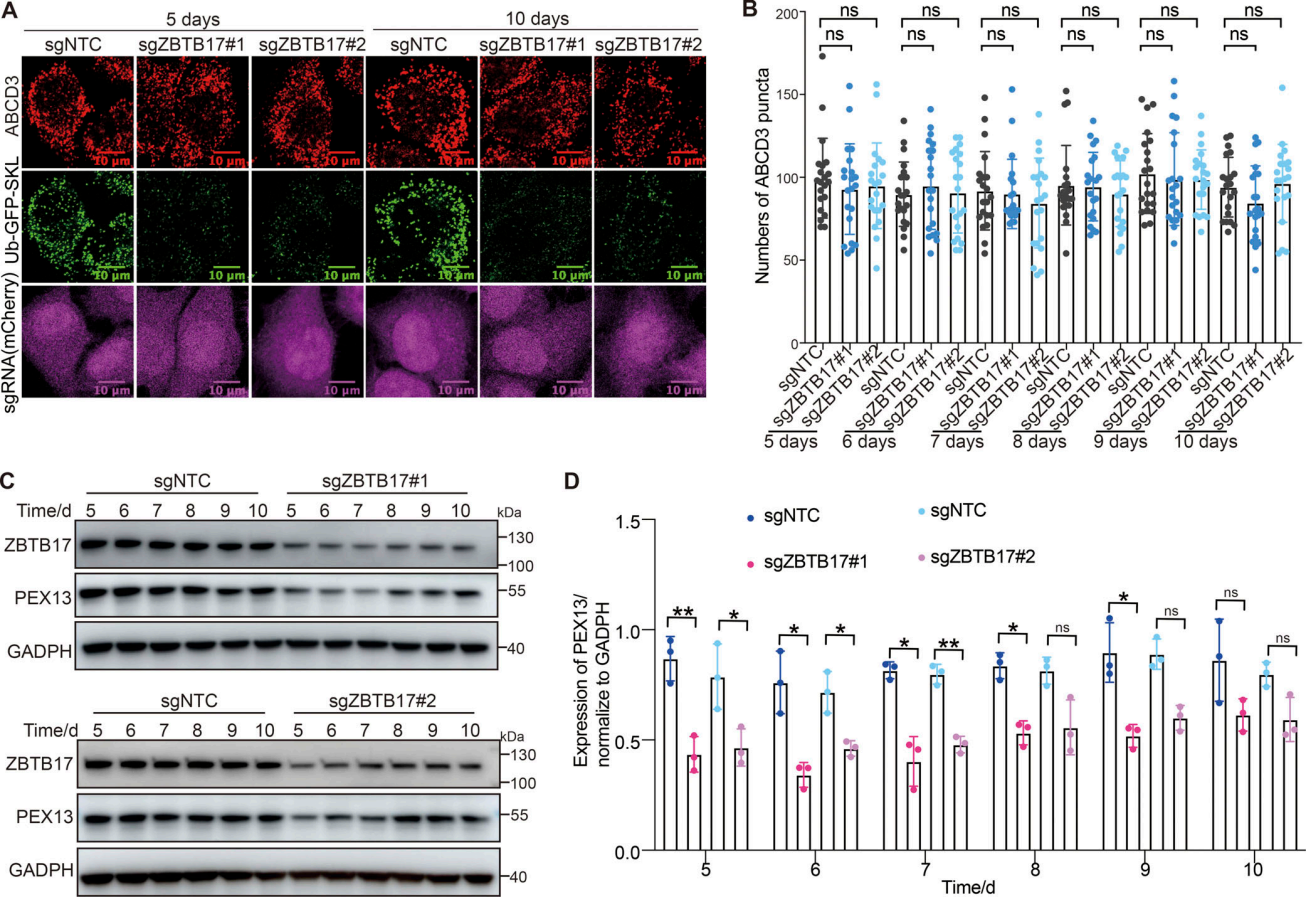
**Peroxisome numbers do not change over time in ZBTB17 knockout cells. (A)** Ub–GFP–SKL HeLa cells were transfected with sgZBTB17 for 10 days and stained for ABCD3. Representative immunofluorescence microscopy images of 5- and 10-day are shown. **(B)** Quantification of peroxisome numbers (ABCD3 puncta) in >20 cells in 5 or 10 days, n.s., not significant (P > 0.05), by two-tailed Student’s *t* test. **(C)** Immunoblotting of PEX13 and ZBTB17 in HeLa cells transfected with sgRNA/Cas9 targeting ZBTB17 for 5–10 days. **(D)** PEX13 levels in C were quantified and normalized using ImageJ. Values are mean ± SD, *n* = 3 independent experiments. *, P < 0.05; **, P < 0.01; n.s., not significant (P > 0.05) by two-tailed Student’s *t* test. Source data are available for this figure: SourceData F6.

A recent study shows that pexophagy is induced in the absence of PEX13 (Demers et al., 2023). We monitored peroxisome numbers and PEX13 protein levels over time (Fig. 6). Pexophagy is usually triggered by the deficiency of peroxisome import. However, peroxisome numbers in *ZBTB17* knockout cells do not change over 10 days, despite the import defect (Fig. 6, A and B). In *ZBTB17* knockout cells, the downregulation of PEX13 is accompanied by the downregulation of peroxisomal enzymes, suggesting less importing stress in ZBTB17-deficient cells than in PEX13-deficient cells. In addition, the PEX13 protein level in ZBTB17 deficient cells is not fully ablated over time (Fig. 6, C and D). We speculate there are other factors involved in PEX13 transcriptional regulation. In fact, it has been reported that ZBTB17 partners with many other transcriptional factors to either enhance or inhibit gene transcription (Aesoy et al., 2014; Wiese et al., 2013). ZBTB17 is a transcriptional regulator that fine-tunes the expression of PEX13 without inducing pexophagy.

### ZBTB17 modulates purine metabolism through its transcriptional regulation of peroxisome import

Peroxisomes are involved in the catabolism of lipids, D-amino acids, polyamines, purines, as well as ROS metabolism. Dysregulation in the import of peroxisome matrix proteins may impact these peroxisome-dependent metabolic pathways. Since PEX13 is regulated by ZBTB17, we wonder if ZBTB17 affects peroxisome-dependent metabolic pathways.

**Figure 7.**
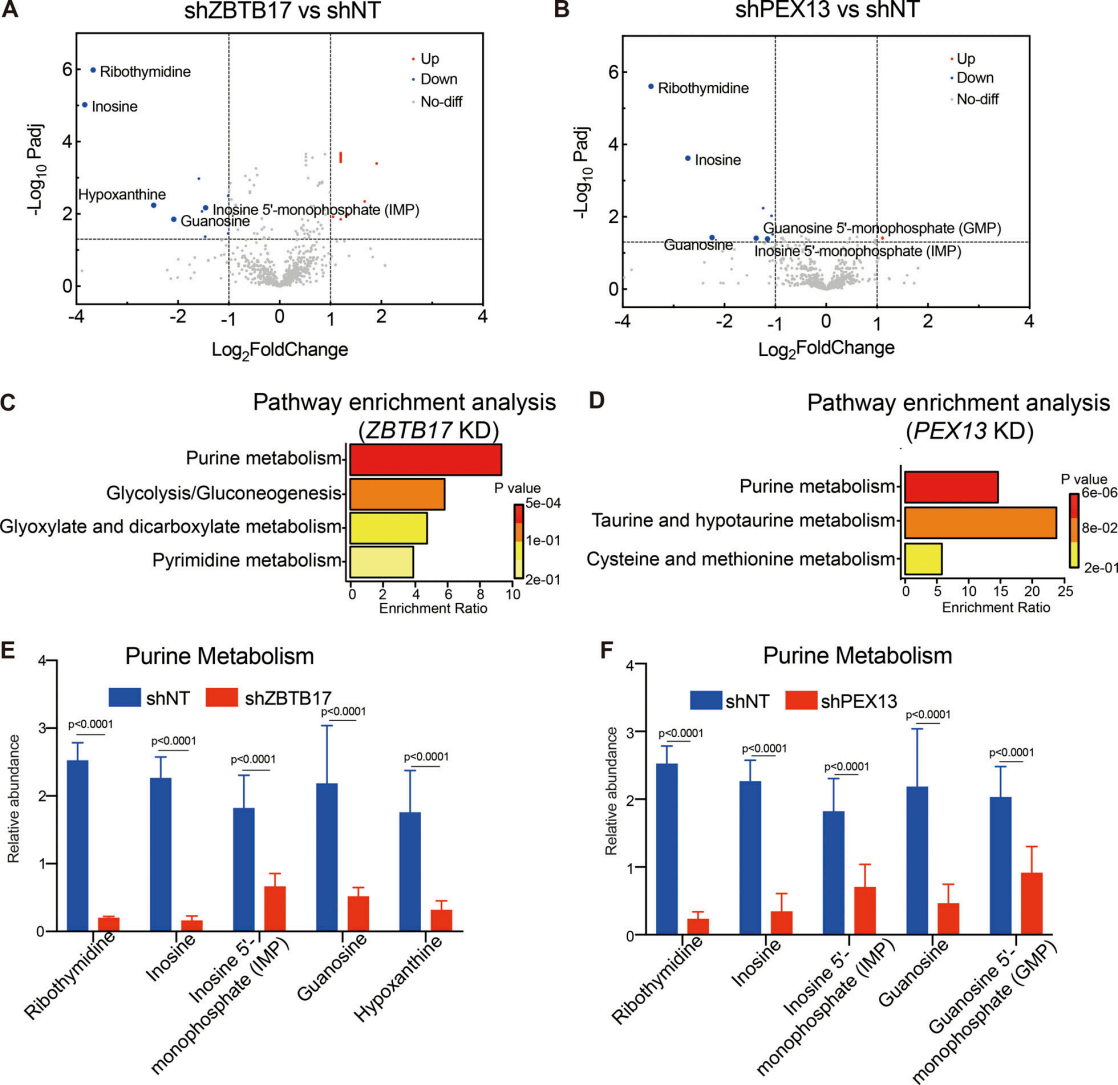
**Metabolic alterations in cells with ZBTB17 or PEX13 knockdown. (A)** The volcano plot of the fold changes of metabolites in cells with or without ZBTB17 knockdown. P values were determined by a two-tailed Student’s *t* test. Blue dots indicate significantly changed metabolites (P < 0.05 and fold change >2, *n* = 6 biologically independent samples in each group). **(B)** The volcano plot of the fold changes of metabolites in cells with or without PEX13 knockdown. P values were determined by a two-tailed Student’s *t* test. Blue dots indicate significantly changed metabolites (P < 0.05 and fold change >2, *n* = 6 biologically independent samples in each group). **(C and D)** Significantly decreased metabolites in ZBTB17 or PEX13 knockdown cells are enriched in the purine metabolism pathway. MetaboAnalyst (https://www.metaboanalyst.ca) was used for pathway enrichment analysis. **(E and F)** Relative abundances of each purine metabolism-related metabolite in cells with ZBTB17 or PEX13 knockdown are illustrated. Values are mean ± SD, *n* = 6 independent samples in each group. P values were determined by the two-way ANOVA multiple comparisons test.

We profiled the metabolomic changes in *ZBTB17* or *PEX13* knockdown cells using liquid chromatography–mass spectrometry (LC–MS)–based untargeted metabolomics. Metabolite annotation was performed using MetDNA (Shen et al., 2019; Zhou et al., 2022), and metabolomics data was normalized to sample protein concentrations. Metabolite peak intensity was used to calculate the fold-changes of metabolites between samples. Compared with the control cells, 8 metabolites in *ZBTB17* knockdown cells and 10 metabolites in *PEX13* knock down cells were significantly downregulated (P < 0.05; and fold change >2) (Fig. 7, A and B; and Table S6). Metabolic pathway enrichment analysis further revealed that the purine metabolism pathway was one of the downregulated pathways in both *ZBTB17* and *PEX13* knockdown cells (Fig. 7, C and D). Ribothymidine, inosine, inosine 5′-monophosphate (IMP), and guanosine were remarkably reduced in both *ZBTB17* and *PEX13* knockdown cells. Hypoxanthine was only decreased in *ZBTB17* knockdown cells, while guanosine 5′- monophosphate (GMP) was decreased in *PEX13* knockdown cells (Fig. 7, E and F). These data show that *ZBTB17* or *PEX13* knockdown causes similar metabolic shifts among the measured metabolites, suggesting that ZBTB17-driven metabolic changes partly arise from reduced PEX13 expression and subsequent peroxisome dysfunction.

## Discussion

We have presented here a focused genetic screen employing the CRISPR/Cas9 system to identify novel regulators of peroxisomes in cells. Our screen has generated a comprehensive dataset that not only reaffirms the role of known peroxisomal genes but also unveils previously unrecognized transcriptional regulation mechanism of peroxisomes, and the related purine metabolism.

Despite the power of the CRISPR-based screening system, our screens suggest the selection strategy matters for peroxisomes. Cell viability and the fluorescence reporter are two major phenotypic readouts. A recent study used cell viability to screen peroxisome regulators and revealed the connection between Wnt signaling and peroxisomes (Vu et al., 2024). In this study, we used a fluorescence reporter, which can be fine-tuned to adapt different screening stringency. In this study, we arbitrarily set the bottom 30% GFP/mCherry expression as the cut-off. One or two rounds of selection were used. While our screen successfully identified the established peroxins PEX2/10/12, ZBTB17 stood out as the only novel candidate showing regulatory effects. The identification of a few regulators was anticipated due to the nature of our pilot screen, which utilized a focused sgRNA library covering just 573 genes. Importantly, this pilot study revealed that the choice of selection parameters plays a crucial role in determining screening effectiveness, providing valuable insights for future larger-scale screens. For future screening of more comprehensive sgRNA libraries, different selection strategies with different fluorescence cut-off or selection rounds should be tested and applied to best fit the needs. In general, a stringent screen is more likely to identify the dominant regulators, while a less stringent screen will enhance the possibility to identify all regulators, and also inevitably increase the chance for false positive enrichment.

The GFP–SKL /mCherry-based screening system measures relative GFP–SKL fluorescent signals that can be influenced by multiple peroxisomal processes, including peroxisome biogenesis, protein import, and peroxisome degradation. In the screening cell line, GFP–SKL predominantly localizes to peroxisomes under normal conditions. However, GFP–SKL would redistribute to the cytosol when peroxisome numbers decrease or protein import is compromised.

A reduction in cellular GFP–SKL signal can result from multiple mechanisms: decreased peroxisome biogenesis, enhanced pexophagy-mediated organelle degradation, or impaired peroxisomal protein import. This complexity arises from the differential degradation kinetics of GFP–SKL in peroxisomal versus cytosolic compartments. For example, *PEX3* knockout, which blocks peroxisome biogenesis and eliminates peroxisomes, causes GFP–SKL to remain cytosolic. Notably, these cells show significantly reduced GFP–SKL fluorescence (Fig. 1 B), indicating that cytosolic GFP–SKL undergoes more rapid degradation compared with its peroxisome-localized counterpart. Knockout or disruption of *PEX2*, *PEX10*, *PEX12*, or *PEX13* impairs peroxisomal protein import, causing GFP–SKL to accumulate in the cytosol where it undergoes faster degradation, ultimately resulting in reduced cellular GFP–SKL signal.

Given these multiple influences on GFP–SKL signals, complementary assays are essential to precisely determine which aspects of peroxisome function are affected. To address this limitation, Ub–GFP–SKL was developed as a more specific reporter for peroxisomal import efficiency. This modified reporter exploits the rapid degradation of cytosolic Ub–GFP–SKL while maintaining the stability of the peroxisome-imported fraction. By amplifying the differential degradation rates between cytosolic and peroxisomal GFP, Ub–GFP–SKL provides a more sensitive and specific readout of peroxisomal import function.

Our screen identified ZBTB17, a previously unrecognized regulator of the peroxisomal protein PEX13, as a positive regulator of peroxisome import. ZBTB17 belongs to the BTB ubiquitin ligase family, characterized by its N-terminal BTB domain. We confirmed the interaction between ZBTB17 and Cullin3, a scaffold protein for cullin-RING ligases (CRLs), and assessed the potential ubiquitin ligase activity of ZBTB17. However, we discovered that the BTB domain is dispensable for ZBTB17-mediated transcriptional regulation of PEX13. The role of ZBTB17 as a transcriptional regulator may have been underestimated, as previous *ZBTB17* knockout mice were created by deleting the BTB/POZ domain (Do-Umehara et al., 2020). Our discovery that ZBTB17 without the BTB domain retains some of its function as a transcription factor implies that different *ZBTB17* knockout strategies should be considered.

PEX13 appears to be a pivotal node in the transcriptional regulation of peroxisome import. Besides PEX13, the expression of several other genes related to peroxisome import was also affected. Notably, PEX2 was downregulated, while PEX14 exhibited upregulation (Fig. S4 C). Despite the upregulation of PEX14, the absence of PEX13 resulted in a restriction of protein import, in line with the indispensable role of PEX13 in protein translocation (Elgersma et al., 1996; Erdmann and Blobel, 1996; Gould et al., 1996). Apart from the genes involved in the peroxisomal import machinery, several peroxisomal proteins, including metabolic enzymes like SCP2, ECH1, and PECR exhibit downregulation (Fig. 4 A). This observation suggests that ZBTB17 may have a broader impact on diverse metabolic pathways within peroxisomes, a dimension not investigated in our current study. It is conceivable that ZBTB17 governs peroxisome function by influencing both protein import and the expression levels of metabolic enzymes.

Peroxisomes are closely associated with purine metabolism as they house three essential enzymes, xanthine dehydrogenase (XDH), Uricase (Uox), and allantoicase (ALLC), involved in purine degradation, located within the peroxisome matrix (Wanders, 2014). However, in human cells, active Uox and ALLC are absent, leading to an inability to further oxidize urate. XDH serves as a crucial enzyme in purine metabolism, catalyzing the oxidation of hypoxanthine to xanthion and xanthion to urate. Defects in peroxisomal import may result in altered distribution of XDH, disturbing the concentration of hypoxanthine and other upstream metabolites in the same pathway, including inosine, inosine 5′-monophosphate (IMP), and guanosine. As a result, it is not surprising that purine metabolism is downregulated in *PEX13* knockdown cells. Our study also demonstrates a similar downregulation of purine metabolism in *ZBTB17* knockdown cells, further corroborating that the metabolomic changes induced by the transcription factor *ZBTB17* are likely related to its regulation of peroxisomal import via *PEX13*. Peroxisomal homeostasis can be regulated through transcription factors such as PPAR family proteins and HIF transcription factors (Lemberger et al., 1996; Walter et al., 2014). We have revealed one more transcriptional regulation mechanism for peroxisome function.

Currently, there is no evidence linking ZBTB17 to peroxisome biogenesis disorders (PBDs). Since ZBTB17 only moderately downregulates PEX13, and this mild downregulation does not trigger pexophagy, it is unlikely that ZBTB17 defects would result in severe PBDs. While ZBTB17’s broader role in human disease remains largely unknown, limited studies indicate its involvement in immune system regulation, including B cell homeostasis, T cell differentiation, and macrophage activation (Piskor et al., 2022; Saba et al., 2011; Zhang et al., 2021). Interestingly, recent research suggests that peroxisome deficiency affects hepatic immune cell development (Parsons et al., 2024). These findings raise the intriguing possibility that ZBTB17 may influence immune cell function through peroxisome regulation.

## Materials and methods

### Cells and reagents

HeLa, HCT116, and HEK293 FT cells were cultured in Dulbecco’s modified Eagle’s medium (C11995500BT-20; Gibco) supplemented with 10% fetal bovine serum (100-500; Gemini), 1% penicillin-streptomycin (15140122; Gibco), and 5% CO_2_ at 37°C.

Antibodies and other reagents used are as follows: anti-Flag (GNI4110-FG-S 1:1,000 WB; GNI), anti-Flag-HRP (GNI4310-FG-S 1:1,000 WB; GNI), anti-HA (2367S 1:1,000 WB; Cell Signaling), anti-HA-HRP (2999S 1:1,000 WB; Cell Signaling), anti-V5 (13202S 1:1,000 WB; Cell Signaling), anti-actin (60008-1-Ig-100ul 1:1,000 WB; Proteintech), anti-GAPDH (AP0063-50ul 1:1,000 WB; Bioworld), anti-Tubulin (AC012 50 µl 1:1,000 WB; ABclonal), anti-PMP70 (P0497-200UL 1:500 IF; Sigma-Aldrich), anti-PMP70 (SAB4200181-200UL 1:1,000 WB; Sigma-Aldrich), anti-Catalase (219010-1ML 1:1,000 WB, 1:500 IF; Merck), anti-PEX13 (sc-271477 1:500 WB; Santa Cruz), anti-ZBTB17 (sc-136985 1:500 WB; Santa Cruz), anti-ZBTB17 (sc-136985X ChIP; Santa Cruz), Donkey Anti-Rabbit IgG H&L (Alexa Fluor 647) (ab150075 1:500 IF; Abcam), Hygromycin (ant-hg-1; Invitrogen), puromycin (ant-pr-1; InvivoGen), and polybrene (40804ES86; Yeasen).

### Generation of the peroxisome reporter GFP–SKL–IRES–mCherry cell line

To generate the peroxisome reporter cell line, we first generated the HeLa cell line stably expressing spCas9. Lentivirus was produced by co-transfecting Cas9–T2A–BFP–Flag plasmid along with two viral packaging plasmids into HEK293 FT cells using Polyethylenimine transfection reagent (9002-98-6; PolySciences). Approximately 48 h after transfection, the viral supernatant was harvested and subsequently filtered through a 0.22 µm filter. Additionally, to increase viral titer, the supernatant was concentrated using PEG virus precipitation reagents (K904-50; BioVision). HeLa cells were transfected with Cas9–T2A–BFP–Flag virus. 2 days after transduction, BFP-positive cells were sorted into a 96 well-plate for single cell clones using a BD FACSAria ΙΙΙ flow cytometer. The single-cell clones were allowed to amplify for 2–3 wk. An immunoblot assay with the anti-Flag antibody was conducted to assess the expression of Cas9, and T7 endonuclease I-cutting assay (M0302S; NEB) was used to examine the activity of spCas9 in the isolated clones. Clone # 3-5-5 was used for future screening experiments. To generate the peroxisome reporter GFP–SKL–IRES–mCherry cell line, spCas9 expressing HeLa cells were transduced with a GFP–SKL–IRES–mCherry lentivirus. Cells expressing both GFP and mCherry were isolated by fluorescence-activated cell sorting using a BD FACSAria ΙΙΙ. Single-cell clones were generated and validated.

### CRISPR library screen

A total of 1.8 × 10^7^ GFP–SKL–IRES–mCherry HeLa cells were transduced on Day 1 with the ubiquitin ligase lenti-CRISPR library (a gift from Haopeng Wang’s lab, ShanghaiTech University, Shanghai, China) at an MOI of 0.3 with an ∼1,000-fold library coverage. 24 h after transduction, hygromycin (600 µg/ml) was added to cells and maintained for 5 days. On Day 7, the surviving cells were sorted for the lowest and highest 30% of the GFP/mCherry ratio by BD FACSAria ΙΙΙ.

To extract genomic DNA, cells were resuspended in 400 µl P1 buffer (19051; Qiagen) with 100 µg/ml RNaseA and 40 µl 10% SDS (S001-100g; MDBio). After incubating at room temperature for 15 min, the lysate was heated at 55°C for 30 min in the presence of Proteinase K (100 µg/ml, P6556-25MG; Sigma-Aldrich). After digestion, samples were passed through a needle of different sizes (21G-23G-25G-27G) multiple times to shear DNA to ∼20 kb size (the criterion for judgment is whether the solution can easily pass through the needle). 400 µl phenol: chloroform: isoamyl alcohol (25:24:1, vol/vol) was added into homogenized samples before centrifugation at 3,000 × *g* for 20 min at room temperature. The aqueous phase was transferred into ultracentrifuge tubes and thoroughly mixed with 40 µl 3 M sodium acetate plus 320 µl isopropanol at room temperature before centrifugation at 12,000 × g for 15 min at 4°C. The gDNA pellets were carefully washed with 1 ml 70% ethanol and centrifuged at 3,000 × g for 15 min. The pellets were dried at 37°C for 30 min and resuspended in water.

Multiple PCR reactions were prepared to amplify the region coding sgRNA from the extracted genomic DNA. For the first round of PCR, the total volume was 100 µl containing 50 µg sheared gDNA, 0.3 µM forward (5′-CTG​CCA​TTT​GTC​TCG​AGG​TCG-3′) and reverse (5′-GCT​CGG​CGC​CAG​TTT​GAA​TAT-3′) primer, 200 µM each dNTP, 1× Titanium Taq buffer, and 1 µl Titanium Taq (639209; Clontech). PCR cycles were 1× (94°C 3 min), 16× (94°C 30 s, 65°C 10 s, 72°C 20 s), and 1× (68°C 2 min). A second round of PCR was performed to add different barcodes for different samples. The total volume of the second round PCR was 100 µl containing 2 µl round 1 PCR product, 0.5 µM forward and 0.5 µM reverse primer, 200 µM dNTP, 5× Prime Star buffer, and 1 µl Prime Star DNA Polymerase (R010A; Takara). PCR cycles were 1× (94°C 3 min), 16× (94°C 30 s, 55°C 10 s, 72°C 20 s), and 1× (68°C 2 min). The main PCR products (∼250 bp) were gel-purified from a 1% agarose gel and submitted for sequencing on an Illumina HiSEq-PE150. Sequencing reads were aligned to the sgRNA library and quantified by MAGeCK (Model-based Analysis of Genome-wide CRISPR-Cas9 Knockout) by following its detailed step-by-step workflow (https://sourceforge.net/p/mageck/wiki/Home/), based on the methodology previously described (Li et al., 2014). A pseudo count was added to each value and the log_2_-transformed fold change in abundance was then calculated between low and high fractions by MaGeCK-test analysis.

Primers used to add barcodes:

Forward: 5′-AAT​GAT​ACG​GCG​ACC​ACC​GAG​ATC​TAC​ACC​GAC​TCG​GTG​CCA​CTT​TT-3′;
Reverse: 5′-CAA​GCA​GAA​GAC​GGC​ATA​CGA​GAT​CCT​GAG​ATT​TCT​TGG​GTA​GTT​TGC​AGT​TTT-3′ (GFP low population) and 5′-CAA​GCA​GAA​GAC​GGC​ATA​CGA​GAT​CGA​CTC​ATT​TCT​TGG​GTA​GTT​TGC​AGT​TTT-3′ (GFP high population).

### Gene knockout and knockdown

To knockout a specific gene in cells with CRISPR/Cas9, sgRNA targeting the specific gene was cloned into MP-783 (Tromp et al., 2018). A lentiviral vector expressed PuroR–T2A–mCherry under the EF1A promoter and a sgRNA sequence under the U6 promoter. MP-783 sgRNAs lentivirus production and infection were performed as described in the above section. 2 days after infection, HeLa cells were treated with puromycin (2 µg/ml) for 3 days for selection. The surviving cells were sorted for mCherry-positive by BD FACSAria ΙΙΙ. The sgRNA sequences used in this study are summarized in Table S1.

To knockdown a specific gene, we used shRNA. The shRNAs targeting a specific gene were cloned into pLKO.1 puro (10878; Addgene) vector using EcoRI (R3101; NEB) and AgeI (R3552S; NEB) restriction sites. HeLa cells were transfected with shRNA lentivirus (shZBTB17, shPEX13, and shNT) for 2 days and then puromycin (2 µg/ml) was added to select for infected cells. The media was changed to fresh puromycin-containing media for 4 days. The sequences of shRNA used in this study are summarized in Table S7.

### Immunofluorescence

HeLa cells were cultured overnight on glass coverslips in DMEM and supplemented with 5% CO_2_ at 37°C. The cells were rinsed three times with 1× phosphate-buffered saline (PBS) and fixed for 15 min at room temperature in 4% paraformaldehyde (MA0192; meilunbio). Cells were permeabilized with 0.1% NP-40 in PBS at room temperature for 15 min. Coverslips were then blocked with 2.5% BSA (36105ES25; Yeasen) in a cell-staining buffer (FXP005; 4A Biotech) for 1 h at room temperature before adding primary antibody stain to coverslips at 4°C overnight. After primary staining, cells were washed three times with PBS and then stained with fluorochrome-conjugated secondary antibodies for 1.5–2 h at room temperature. After staining, coverslips were incubated with DAPI for 10 min and then adhered to a microscopy slide using Mounting Medium (H-1000; Vectorlabs). Coverslips were imaged on a Zeiss LSM 980 Airyscan2 (objective ×40 1.30 NA OIL).

### Immunoblot and immunoprecipitation

Cells were homogenized in lysis buffer (50 mM Tris, 200 mM NaCl, 1% NP-40, pH 7.5) with protease inhibitors (B14001; Biomake) for 30 min at 4°C. The lysates were centrifuged at 13,000 × *g* for 10 min at 4°C to remove debris and to collect the whole-cell extract. The supernatant was subjected to BCA Protein Assay (PA115-01; TIANGEN) to quantify protein levels. After boiling the sample with 1x Protein Loading Buffer (DL101-02; TransGen Biotech) at 95°C for 10 min, equal amounts of proteins were loaded and separated on a 4–20% SDS–PAGE (M42015C; GenScript). The proteins were transferred to a PVDF membrane (10600023; Cytiva) and incubated with specific primary antibodies. The primary antibody signal was visualized by HRP-conjugated to the corresponding secondary antibodies and the ImageQuant system (AI680UV; GE Imager).

For immunoprecipitation with Flag-tagged Cul3, HEK293T cells were homogenized in lysis buffer (50 mM Tris, 200 mM NaCl, 1% NP-40, pH 7.5) with protease inhibitors (B14001; Biomake) for 30 min at 4°C. After removing debris, 20 µl of anti-Flag magnetic beads (B26101; Bimake) were added and the mixture was incubated on a rotator at 4°C overnight. The beads were precipitated using a magnetic stand, washed with cold lysis buffer five times, resuspended in 3x protein loading buffer, and boiled at 95°C for 10 min. The beads were centrifuged at 12,000 rpm for 5 min before SDS–PAGE analysis. The enriched proteins were analyzed by immunoblot (IB).

### Ubiquitination assay

HEK293T cells were transfected with HA-Ub, ZBTB17-Flag, and Cul3-V5 plasmids. 36 h after transfection, cells were treated with 10 µM MG132 (13697-1; Cayman) for 4 h. Cells were harvested and washed three times with cold PBS. 100× protease inhibitor cocktail (B14001; Biomake) was added to the lysis buffer prior to use. Cell pellets were resuspended in 90 µl lysis buffer (20 mM Tris, 150 mM NaCl, 1% Triton X-100, pH7.5) on ice for 1 h. 10 µl 10% SDS was added to the lysate before sonication. After sonication, the lysate was diluted with lysis buffer to a final concentration of 0.1% SDS. Lysates were centrifuged at 4°C at 13,000 × *g* for 10 min. The supernatant was mixed with 20 µl of Anti-Flag magnetic beads (B26101; Bimake) and incubated on a rotator at 4°C overnight. The beads were washed with cell lysis buffer containing 500 mM NaCl five times and heated in 3× denaturing loading buffer for 10 min at 95°C before being resolved by SDS–PAGE.

### RNA extraction and quantitative PCR

500 µl RNAiso Plus (9109; Takara) was added to 0.5 × 10^6^ cells with immediate mixing. The mixture was then added to 100 µl Trichloromethane and inverted for 15 s. After 10 min incubation, centrifuged at 4°C at 13,000 × *g* for 10 min. 200 µl of supernatant was added to a 1.5-ml RNA-free tube and then mixed with an equal volume of isopropanol. The mixture was incubated for 15 min and centrifuged at 4°C at 13,000 × *g* for 10 min. The supernatant was removed and added to 1 ml of 70% ethanol and centrifuged at 13,000 × *g* for 10 min at 4°C. The dry pellets were resuspended in RNA-free water and the RNA concentration was adjusted to 100 ng/µl.

RNA was reverse-transcribed using the PrimeScript RT reagent Kit (RR037A; Takara) and the cDNA was used for qPCR using the TB Green Premix Ex Taq (RR420A; Takara) following the supplied protocol. The PCR was run in a QuantStudio 7 Real-Time PCR system. Samples were normalized to Rpl13a gene expression. Primer sequences used in qRT-PCR are presented in Table S8.

### RNA-seq and ChIP-seq

For RNA-seq, three independent biological replicates of experiments were performed. Cells were sent for RNA-seq (Majorbio company). The library was prepared with Illumina TruseqTM RNA sample prep kit and End Repair Mix adaptor sequences were used. Sequencing was performed with Illumina HiSeq. Gene expression profile changes between control shNT cells and shZBTB17 cells were analyzed using Gene Cluster Analysis and KEGG pathways that were differentially regulated in the absence of functional peroxisomes.

HeLa wild-type cells were prepared for ChIP-seq with GENFUND. Cells were crosslinked with 1% formaldehyde (252549; Sigma-Aldrich) for 10 min at room temperature and quenched with 125 mM glycine (A610235; Sangon Biotech). The fragmented chromatin fragments were precleared and then immunoprecipitated with Protein A + G Magnetic beads (16-663; Milliproe) coupled with ZBTB17 antibody. After reverse crosslinking, ChIP and input DNA fragments were end-repaired and A-tailed using the NEBNext End Repair/dA-Tailing Module (E7442; NEB) followed by adaptor ligation with the NEBNext Ultra Ligation Module (E7445; NEB). The DNA libraries were amplified for 15 cycles and sequenced using Illumina NextSeq 500 with single-end 1 × 75 as the sequencing mode. For data analysis, raw reads were filtered to obtain high-quality clean reads by removing sequencing adapters, short reads (length <50 bp), and low-quality reads using Cutadapt (v2.4) (Bolger et al., 2014) and Trimmomatic (v0.35) (Martin, 2011). Then FastQC (v0.11.5) was used to ensure high reads quality. The clean reads were mapped to the human genome (assembly GRCh38) using the Bowtie2 (v2.1.0) (Langmead and Salzberg, 2012) software. Peak detection was performed using the MACS (v2.1.5) (Zhang et al., 2008) peak finding algorithm with 0.005 set as the P value cutoff. Annotation of peak sites to gene features was performed using the ChIPseeker R package (Yu et al., 2015).

### ChIP-qPCR assay

The ChIP assay was performed with anti-Flag (14793S; Cell Signaling) antibodies using a commercial ChIP kit (P2083S; Beyotime). HeLa cells were transfected with lenti-virus of ZBTB17(WT)- 3x Flag, ZBTB17(∆BTB)- 3x Flag, ZBTB17(∆ZNF12)- 3x Flag, or mCherry-3x Flag for 72 h. 270 µl of formaldehyde (37%) was added directly to 10 ml of culture medium to a final concentration of 1% and cells were incubated at 37°C for 10 min. IgG (ab172730; Abcam) was used as mock control. The sequences of ChIP-qPCR primers are listed:

O1(−346 to −238): Forward, 5′-GGA​AGA​CTA​AAG​ACA​ACG​CAC​CT-3′;
Reverse, 5′-AGC​TCA​CTT​AGT​CCT​AGC​GAG​A-3′;
O2(−7 to +136): Forward, 5′-AGA​GCG​TGT​TTC​TTC​CTA​CAA​A-3′;
Reverse, 5′-ACC​TGG​AGC​GTA​AGA​CAC​AAC-3′;
O3(+111 to +214): Forward, 5′-GCC​CGT​TGT​GTC​TTA​CGC​TCC-3′;
Reverse, 5′-GCG​GCT​GGG​ACG​CCA​TCT-3′;

### Luciferase assay

Luciferase reporter vectors were constructed based on the pGL4.10 (E6651; Promega), a vector without enhancer or promoter elements. To produce vectors containing various putative PEX13 promoter motifs, different DNA fragments were inserted at the BglI site upstream of the Firefly luciferase gene. For luciferase assays, 0.8 × 10^4^ HEK293T cells were cotransfected using PEI with plasmids at a ratio of 5 ng Renilla luciferase (pGL4.74), 50 ng pGL4.10 empty vector or pGL4.10-PEX13 reporter, and 50 ng transcription factor expression vector (pEF6a-ZBTB17 or pEF6a empty vector) for 24 h. Luciferase assays were performed in 96-well plates using the Dual-Luciferase Reporter Assay (E1960; Promega) and results were quantified using a luminescence counter (SpectraMax i3). Firefly luciferase activity values were normalized to Renilla luciferase activity.

### Subcellular fractionation

5 × 10^6^ HeLa cells were harvested and washed with PBS. 500 µl peroxisome extraction buffer (5 mM MOPS, pH 7.65, with 0.25 M sucrose, 1 mM EDTA, and 0.1% ethanol, Protease Inhibitor Cocktail) was added to the cell pellet to achieve an even suspension. The suspended cells were homogenized in a 2-ml Dounce homogenizer (D8938-1SET; Sigma-Aldrich) using Pestle B with ∼50 strikes. The sample was centrifuged at 800 × g for 15 min at 4°C. The supernatant was subjected to the BCA Protein Assay (PA115-01; TIANGEN) to quantify protein levels and the same amount of proteins from each sample were centrifuged at 2,300 × g for 15 min at 4°C to obtain the pellet. The supernatant was transferred to a new centrifugation tube and centrifuged at 23,000 × g for 20 min at 4°C. After centrifugation, the pellet (peroxisome fraction) and supernatant (cytosolic fraction) were harvested for further analysis.

### Catalase activity assay

Catalase activity was conducted with peroxisome and cytosolic fraction respectively by using the CheKine Catalase (CAT) Activity Assay Kit (KTB1040; Abbkine). 20 µl samples from above were subjected to catalase activity assay as described in the instruction by measuring A_540_ using a SpectraMax i3 microplate Reader.

### Metabolites extraction and metabolomics

HeLa cell samples (shZBTB17, shPEX13, and shNT) were placed in a 6-cm dish and extracted using a metabolite extraction solution MeOH: ACN: H_2_O (2:2:1, vol/vol) solvent mixture. The dishes were placed on dry ice, 1 ml of cold solvent was added to each cell plate, and the plates were incubated at −80°C for 40 min. The samples were then scraped and transferred to a 1.5-ml EP tube, vortexed for 1 min, and sonicated for 10 min in an ice-bath. To precipitate proteins, samples were centrifuged at 13,000 rpm for 10 min at 4°C. The resulting supernatant was taken and evaporated to dryness in a vacuum concentrator. The dry extracts were then reconstituted in 100 µl of ACN: H_2_O (1:1, vol/vol), sonicated for 10 min, and centrifuged at 13,000 rpm for 15 min at 4°C to remove insoluble debris. The supernatant was then transferred to HPLC vials and kept at −80°C until LC–MS analysis. For each condition, six biological repeats were used. For metabolomics, samples were acquired using a UHPLC system (Vanquish; Thermo Fisher Scientific) coupled to an orbitrap mass spectrometer (Exploris 480; Thermo Fisher Scientific). A Waters BEH amide column was used for LC separation. Mobile phases, linear gradient eluted, and ESI source parameters followed the previous publication (Wang et al., 2022). Metabolite annotation was performed using MetDNA (Shen et al., 2019; Wang et al., 2022; Zhou et al., 2022). Metabolomics data was normalized to sample protein concentrations. Metabolic pathway-enrichment analysis was performed via hypergeometric test and visualized in R (v 4.3.0). The pathway database was KEGG (https://www.genome.jp/kegg/).

### Online supplemental material

Fig. S1 shows the validation of the reporter cell line; Fig. S2 shows the effect of other candidate genes; Fig. S3 illustrates the fractionation experiments showing catalase distribution in different cells. Fig. S4 shows the expression of other peroxisome-related genes; Fig. S5 shows that ZBTB17 regulates the translocation of peroxisomal matrix proteins in HCT116 cells. Table S1 includes the sgRNA sequences used in this study; Table S2 shows the sgRNA sequences of the E3 library; Table S3 shows the complete list of sgRNAs or genes with ranking, related to Fig. 1, D and E; Table S4 shows the differential expression of genes with ranking, related to Fig. S2, A and B and Table S5 show the differentially expressed genes in ZBTB17 KD cells; Table S6 is the metabolomic identifications; Table S7 is the shRNA sequences used in the study; and Table S8 is the summary of primer sequences used for quantitative PCR.

## Supplementary Material

Table S1 includes the sgRNA sequences used in this study.

Table S2 shows the sgRNA sequences of the E3 library.

Table S3 shows the complete list of sgRNAs or genes with ranking, related to Fig. 1, D and E.

Table S4 shows the differential expression of genes with ranking, related to Fig. S2, A and B.

Table S5 shows the differentially expressed genes in ZBTB17 KD cells.

Table S6 is the metabolomic identifications.

Table S7 is the shRNA sequences used in the study.

Table S8 is the summary of primer sequences used for quantitative PCR.

SourceData F3 is the source file for Fig. 3.

SourceData F5 is the source file for Fig. 5.

SourceData F6 is the source file for Fig. 6.

SourceData FS1 is the source file for Fig. S1.

SourceData FS3 is the source file for Fig. S3.

SourceData FS5 is the source file for Fig. S5.

## Data Availability

The RNA sequencing data and ChIP-sequencing data have been deposited in the NCBI Gene Expression Omnibus (GEO) under the accession number GSE239792. All other data are available in the article itself and its online supplementary materials.
