# Supplementary material for: ZBTB17/MIZ1 promotes peroxisome biogenesis by transcriptional regulation of PEX13
Source: J Cell Biol. 2025 Apr 17;224(6):e202407198. doi: 10.1083/jcb.202407198 (PMC12005116; doi:10.1083/jcb.202407198)
Supplement: Table S1 — includes the sgRNA sequences used in this study. [file jcb_202407198_tables1.docx]

**Supplementary Table 1.** The sgRNA sequences used in this study.

| **Gene Name** | **sequence** | **sg#** |
| --- | --- | --- |
| ZBTB17 | AGGGCCAGCTGAGGTCAAGG | sg1 |
|  | GGAGCAAGAGGAGCAAGAGG | sg2 |
| PEX3 | ATTAAGGCCTCTCTCAGTGT | previously validated (PMID: 34747980) |
| UBE4B | GGAAGCCACGCTGCAGAACATGG | sg1 |
|  | GAGCTCTGTGAAACCAAGTTTGG | sg2 |
| ZBTB10 | GGGCTGCGGAGGCCAAGCTG | sg1 |
|  | GGCATGATGAGCTTTCGCGA | sg2 |
| RNF25 | AAAGTGATGTAGATCTCCCA | sg1 |
|  | TGAATCCTGGTCCTCTGCAG | sg2 |
| RNF215 | GCCACGGCTGAGATCACCAG | sg1 |
|  | GTAGTCCAGGCACACCGCAC | sg2 |
| KCNS2 | GAGCCTGTGGGACGTGTCGG | sg1 |
|  | GATAGAAATGCAGCACGTAG | sg2 |
| PHRF1 | GGAACGTGTTACAGAAGCCA | sg1 |
|  | GATGTCATCATCCACCGCGA | sg2 |
| GTF2H2C | GAGTCTGCATAGCCATGCTT | sg1 |
|  | TGGATGGCAATACTGAGCCA | sg2 |
| ASB7 | TGTGGCTGCCCACTACGGCA | sg1 |
|  | TGTTGGCATTGTGGTCCAGG | sg2 |
| PEX2 | TCAAAGCGAGCTAACAGCCC | sg1 |
|  | AGTGCTAAGAATAAGCCAGT | sg2 |
| NTC | ACCGACGTTTAATCGAGGCT | sg1 |
|  | GTAGAACGAGCCAACCATTT | sg2 |
