# Supplementary material for: ZBTB17/MIZ1 promotes peroxisome biogenesis by transcriptional regulation of PEX13
Source: J Cell Biol. 2025 Apr 17;224(6):e202407198. doi: 10.1083/jcb.202407198 (PMC12005116; doi:10.1083/jcb.202407198)
Supplement: Table S7 — is the shRNA sequences used in the study. [file jcb_202407198_tables7.docx]

**Supplementary Table 7.** The shRNA sequences used in this study

| **Gene Name** | **sequence** | **sh#** |
| --- | --- | --- |
| ZBTB17 | Forward：CCGGGTGTTCACTTTAAGGCTCATACTCGAGTATGAGCCTTAAAGTGAACACTTTTT | sh1 |
|  | Reverse：AATTAAAAAGTGTTCACTTTAAGGCTCATACTCGAGTATGAGCCTTAAAGTGAACAC |  |
|  | Forward：CCGGGTGTTCACTTTAAGGCTCATACTCGAGTATGAGCCTTAAAGTGAACACTTTTT | sh2 |
|  | Reverse：AATTCAAAAACGAGAGCTCGGAGCAAGAAATCTCGAGATTTCTTGCTCCGAGCTCTCG |  |
| PEX13 | Forward：CCGGGTGTTCACTTTAAGGCTCATACTCGAGTATGAGCCTTAAAGTGAACACTTTTT | sh1 |
|  | Reverse：AATTCAAAAACCATTGGGAAAGATGGAGAAACTCGAGTTTCTCCATCTTTCCCAATGG |  |
|  | Forward：CCGGGTGTTCACTTTAAGGCTCATACTCGAGTATGAGCCTTAAAGTGAACACTTTTT | sh2 |
|  | Reverse：AATTCAAAAACGGTGGAATCAAGTAAAGTTTCTCGAGAAACTTTACTTGATTCCACCG |  |
