# Supplementary material for: ZBTB17/MIZ1 promotes peroxisome biogenesis by transcriptional regulation of PEX13
Source: J Cell Biol. 2025 Apr 17;224(6):e202407198. doi: 10.1083/jcb.202407198 (PMC12005116; doi:10.1083/jcb.202407198)
Supplement: Table S8 — is the summary of primer sequences used for quantitative PCR. [file jcb_202407198_tables8.docx]

**Supplementary Table 8.** Summary of primer sequences for quantitative PCR

| **Gene Name** | **sequences** | |
| --- | --- | --- |
|  | **Forward** | **Reverse** |
| PEX26 | CTCTGGGCTGCTTATCGGAG | TGAAGTACATCCAGTCGCCG |
| PEX3 | AATTCCGAGAGCCTCACAGC | GGACCCGCAAAAGAACAACC |
| PEX5 | TGGGAGTCCTTTTCAACCTG | CGCCTAGCTTATTCCACAGC |
| PEX12 | TCATTGACTGCCCTGCCTAC | TCACCCGGGTTTTACGACAC |
| PEX19 | GGCTCGTTTTGAGATGGTGCT | AAAGTTGAGGCCAGGAGGCAT |
| PEX2 | GCAGCTAGTTTGGTCCCAGT | TGGCTCAAAGCGAGCTAACA |
| PEX7 | GAGTTCTCCCCGTACCTGCC | TAGTAGGGTTCCACAGCCCG |
| PEX14 | GCCACGGCAGTGAAGTTTCTA | GCTGGAAGGCCATATCAATCTC |
| PEX13 | GGGCCCCACTTTCCAATCTG | TACACGGAGGCGGTTGTAGC |
| ZBTB17 | AGTGTGGGAAGCAGTTCACC | GGACTGGACGA ATCTCTTGC |
| Rpl13a | CGAGGTTGGCTGGAAGTACC | CTTCTCGGCCTGTTTCCGTAG |
