# Supplementary figures and images for: ZBTB17/MIZ1 promotes peroxisome biogenesis by transcriptional regulation of PEX13
Source: J Cell Biol. 2025 Apr 17;224(6):e202407198. doi: 10.1083/jcb.202407198 (PMC12005116; doi:10.1083/jcb.202407198)

**B**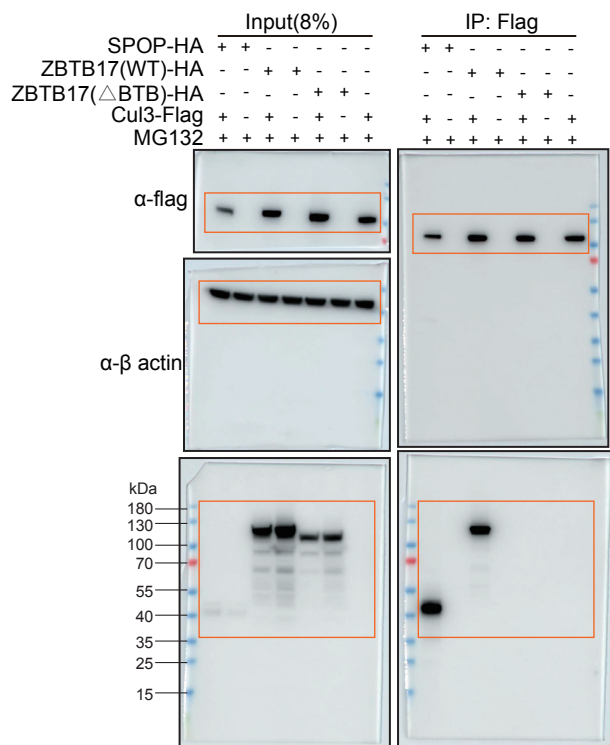**C**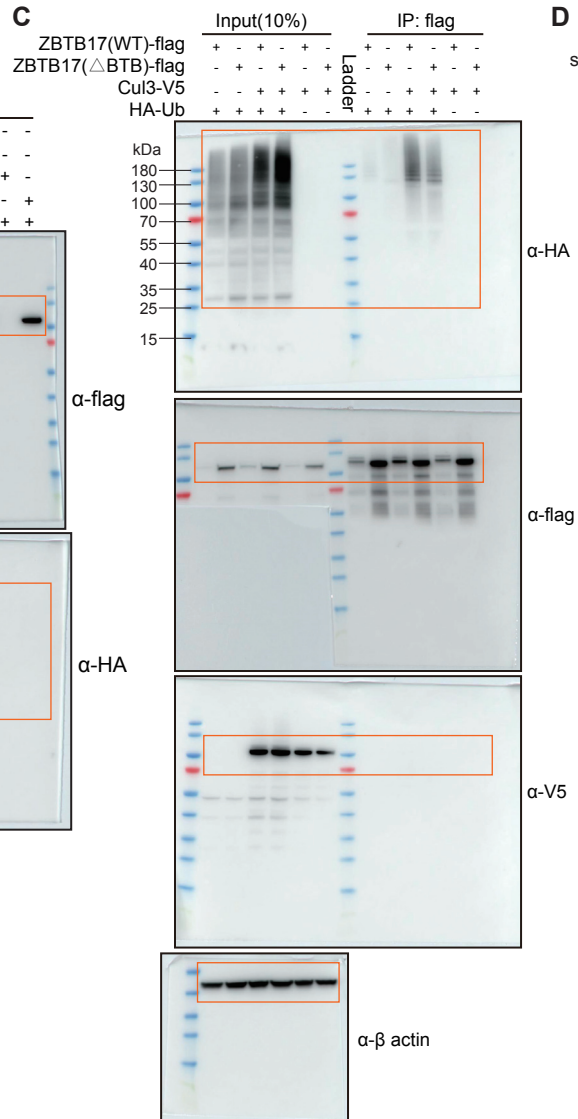**D**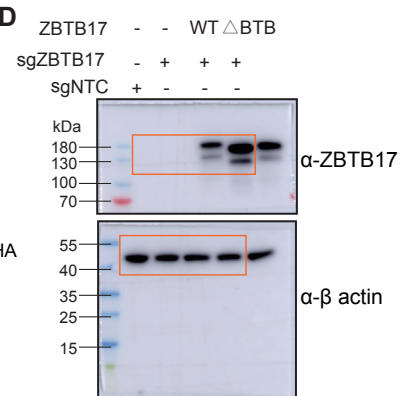

Supplement: SourceData F3 — is the source file for Fig. 3. [file jcb_202407198_sourcedataf3.pdf]

**A**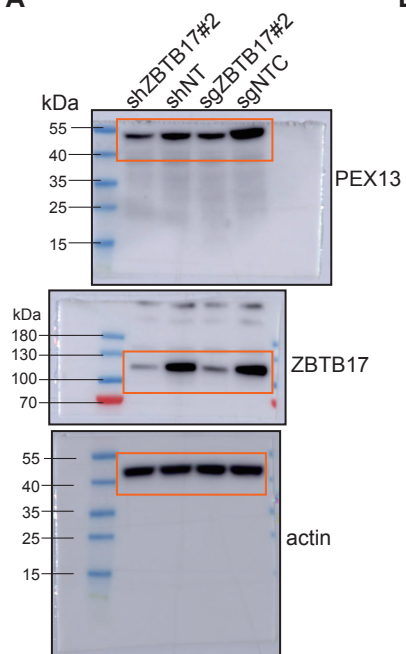**B**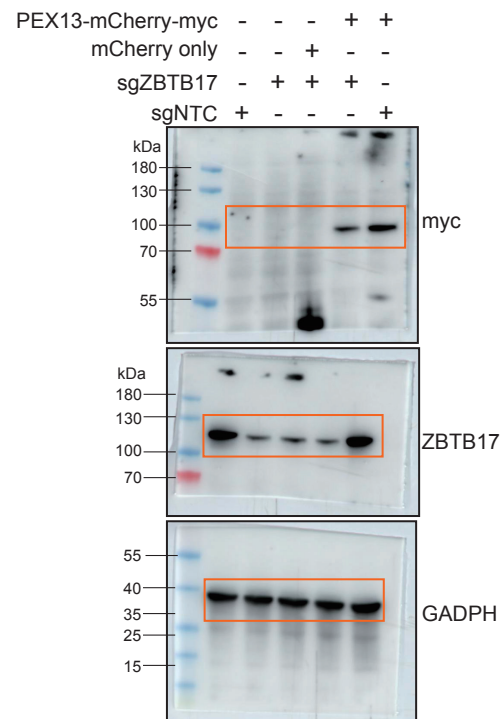

Supplement: SourceData F5 — is the source file for Fig. 5. [file jcb_202407198_sourcedataf5.pdf]

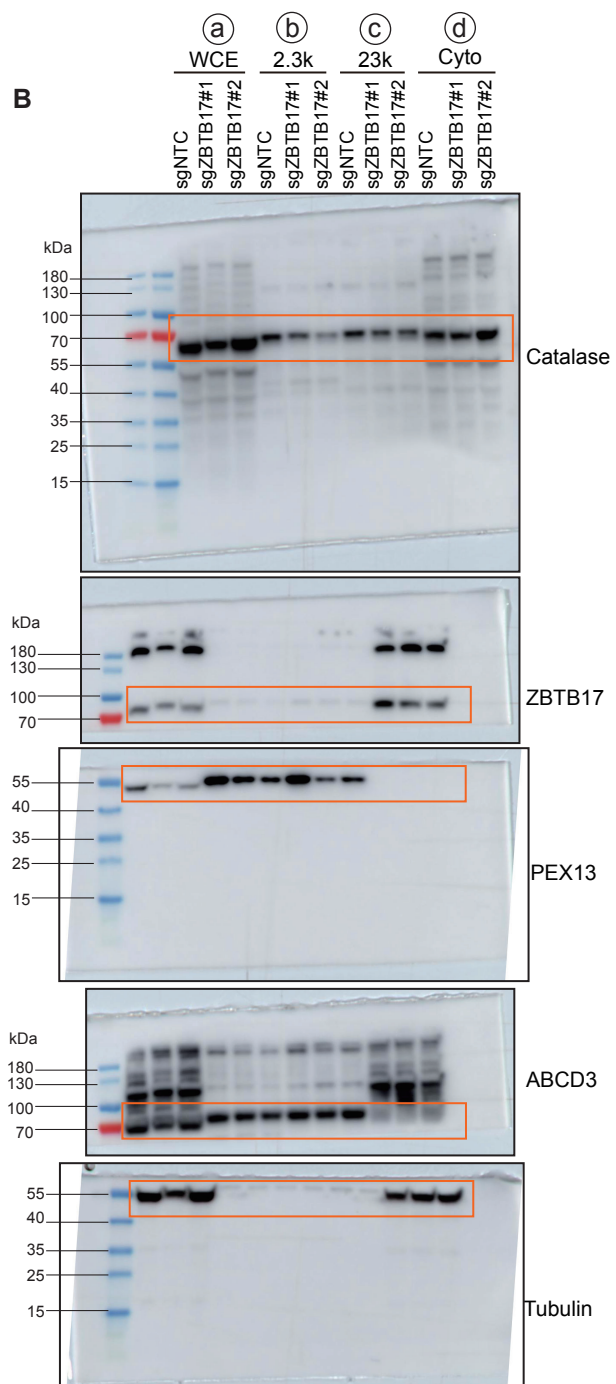

Supplement: SourceData FS3 — is the source file for Fig. S3. [file jcb_202407198_sourcedatafs3.pdf]

**B**

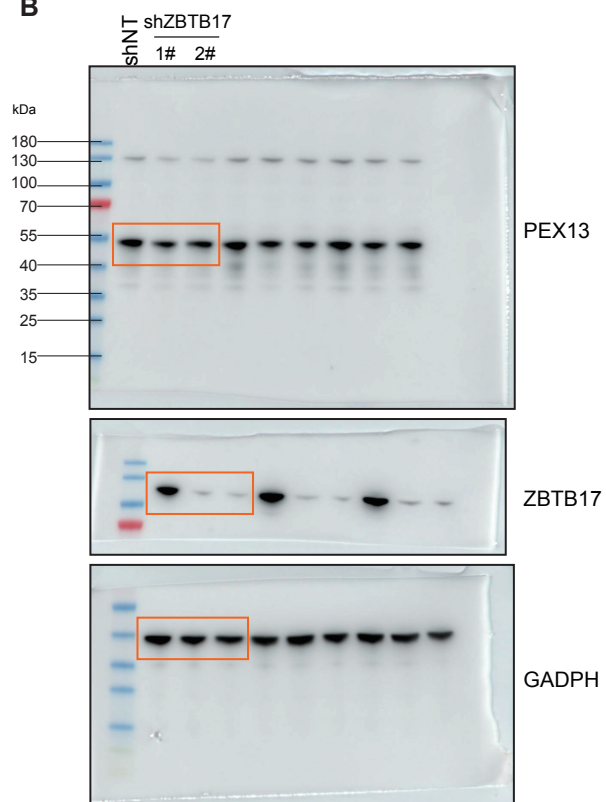

Supplement: SourceData FS5 — is the source file for Fig. S5. [file jcb_202407198_sourcedatafs5.pdf]
