# Supplementary material for: ZBTB17/MIZ1 promotes peroxisome biogenesis by transcriptional regulation of PEX13
Source: J Cell Biol. 2025 Apr 17;224(6):e202407198. doi: 10.1083/jcb.202407198 (PMC12005116; doi:10.1083/jcb.202407198)
Supplement: SourceData FS1 — is the source file for Fig. S1. [file jcb_202407198_sourcedatafs1.pdf]

A

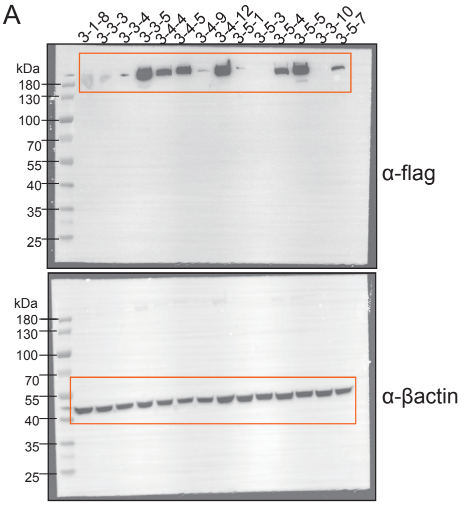
3-1-8
3-3-3
3-3-4
3-3-5
3-4-4
3-4-5
3-4-9
3-4-12
3-5-1
3-5-3
3-5-4
3-5-5
3-3-10
3-5-7
kDa
180
130
100
70
55
40
35
25
α-flag
kDa
180
130
100
70
55
40
35
25
α-βactin

B

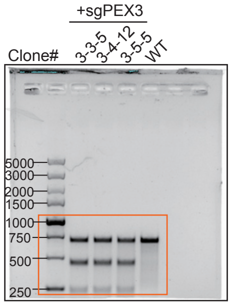
+sgPEX3
Clone#
3-3-5
3-4-12
3-5-5
WT
5000
3000
2000
1500
1000
750
500
250
