# Supplementary material for: ZBTB17/MIZ1 promotes peroxisome biogenesis by transcriptional regulation of PEX13
Source: J Cell Biol. 2025 Apr 17;224(6):e202407198. doi: 10.1083/jcb.202407198 (PMC12005116; doi:10.1083/jcb.202407198)
Supplement: SourceData F6 — is the source file for Fig. 6. [file jcb_202407198_sourcedataf6.pdf]

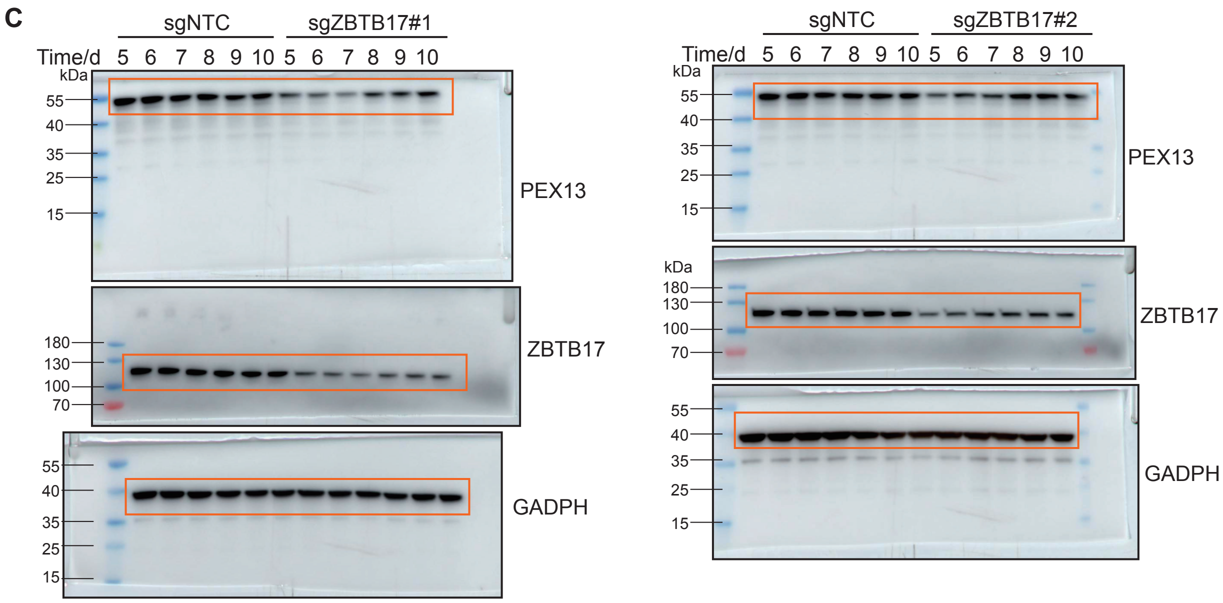
C
sgNTC
sgZBTB17#1
Time/d 5 6 7 8 9 10 5 6 7 8 9 10
kDa
55
40
35
25
15
PEX13
180
130
100
70
ZBTB17
55
40
35
25
15
GADPH
sgNTC
sgZBTB17#2
Time/d 5 6 7 8 9 10 5 6 7 8 9 10
kDa
55
40
35
25
15
PEX13
kDa
180
130
100
70
ZBTB17
55
40
35
25
15
GADPH
